# Early nucleation events in the polymerization of actin, probed by time-resolved small-angle x-ray scattering

**DOI:** 10.1038/srep34539

**Published:** 2016-10-24

**Authors:** Toshiro Oda, Tomoki Aihara, Katsuzo Wakabayashi

**Affiliations:** 1 X-ray Structural Analysis Research Team, RIKEN SPring-8 Center, RIKEN Harima Institute, Kouto 1-1, Sayo, Hyogo 679-5148, Japan; 2 Graduate School of Engineering Science, Osaka University, Toyonaka, Osaka 560-8531, Japan

## Abstract

Nucleators generating new F-actin filaments play important roles in cell activities. Detailed information concerning the events involved in nucleation of actin alone *in vitro* is fundamental to understanding these processes, but such information has been hard to come by. We addressed the early process of salt-induced polymerization of actin using the time-resolved synchrotron small-angle X-ray scattering (SAXS). Actin molecules in low salt solution maintain a monomeric state by an electrostatic repulsive force between molecules. On mixing with salts, the repulsive force was rapidly screened, causing an immediate formation of many of non-polymerizable dimers. SAXS kinetic analysis revealed that tetramerization gives the highest energetic barrier to further polymerization, and the major nucleation is the formation of helical tetramers. Filaments start to grow rapidly with the formation of pentamers. These findings suggest an acceleration mechanism of actin assembly by a variety of nucleators in cells.

Some motility systems in eukaryotic cells are driven by dynamic cycles of polymerization-depolymerization of actin. Actin binding proteins help the microscopic cycles to be efficiently combined into the macroscopic activities of cells^1^,^2^. Among actin binding proteins, nucleators such as an Arp2/3 complex^3^ and formin^4^ catalyze the initial formation of filament “seeds” consisting of multiple actin subunits^5^. The activities of nucleators are controlled by cofactors or nucleation-promoting factors, and thereby new filaments are generated in places to be required and at the proper timing in cells. Since 2000, many nucleators have been newly identified, such as Spire^6^, Cordon-bleu^7^, Leiomodin^8^, p53-cofactor JMY^9^ and adenomatous polyposis *coli*^10^. These nucleators can be classified into several groups by their unique structures and individual seeding mechanisms^11^. Comparison of the actions of nucleators with the events in the nucleation stage of actin molecules alone, without additional nucleators, would provide us a more complete, underlying picture of actin filament generation induced by nucleators in cells.

*In vitro*, G-actin prepared in low salt solution is spontaneously polymerized into F-actin by adding neutral salts^12^. In 1962, Oosawa and Kasai^13^ proposed a mechanism for actin polymerization as a condensation phenomenon, similar to a vapor-liquid equilibrium, which includes an initial nucleation step, followed by subsequent growth from the nuclei. The nucleation-growth model for the salt-induced polymerization of actin has been kinetically confirmed by monitoring the fraction of F-actin formed as a function of time during polymerization^14^,^15^,^16^. In many classical studies, the simplifying assumption that a nucleus is in steady-state equilibrium with monomers and that the nucleus grows into the filament at the same rate constant as filament elongation has been used, and the nucleation process is treated using a single overall dissociation constant^14^. Hence, these studies have provided no information regarding the actual pathway for the formation of nuclei. Current studies on the polymerization of actin have provided the mechanisms, which describe the process quantitatively^17^,^18^, but the nature of the nucleation process that initiates actin polymerization remains rather elusive. In this article, we characterize early processes of actin polymerization by using the time-resolved synchrotron small-angle X-ray scattering (SAXS) and a stopped flow mixer. Time-dependent SAXS intensity data from various concentrations of actin are analyzed to identify a plausible pathway of oligomeric intermediates formed in the early stage of polymerization. Our SAXS kinetic analysis shows that actin polymerization under the present conditions is a nucleation-controlled reaction: after initial dimerization including non-polymerizable dimers, subsequent trimerization from dimers and monomers yields a large energetic barrier to further polymerization, but tetramerization needs additional energy as a nucleating process. Evidence is provided, however, that it is pentamers that serve as “seed” for further polymerization of actin. In this article, “nucleus” is defined as the least stable intermediate in the pathway of polymerization^19^,^20^, and “seed” is termed as a stable oligomeric intermediate with a minimum length of F-actin from which the canonical elongation of actin is fostered. Finally, the results are briefly discussed in relation to the requirement of nucleator to generate new filaments in cells.

## Results

### SAXS intensity profiles from G-actin in G-buffer

**Figure 1:**
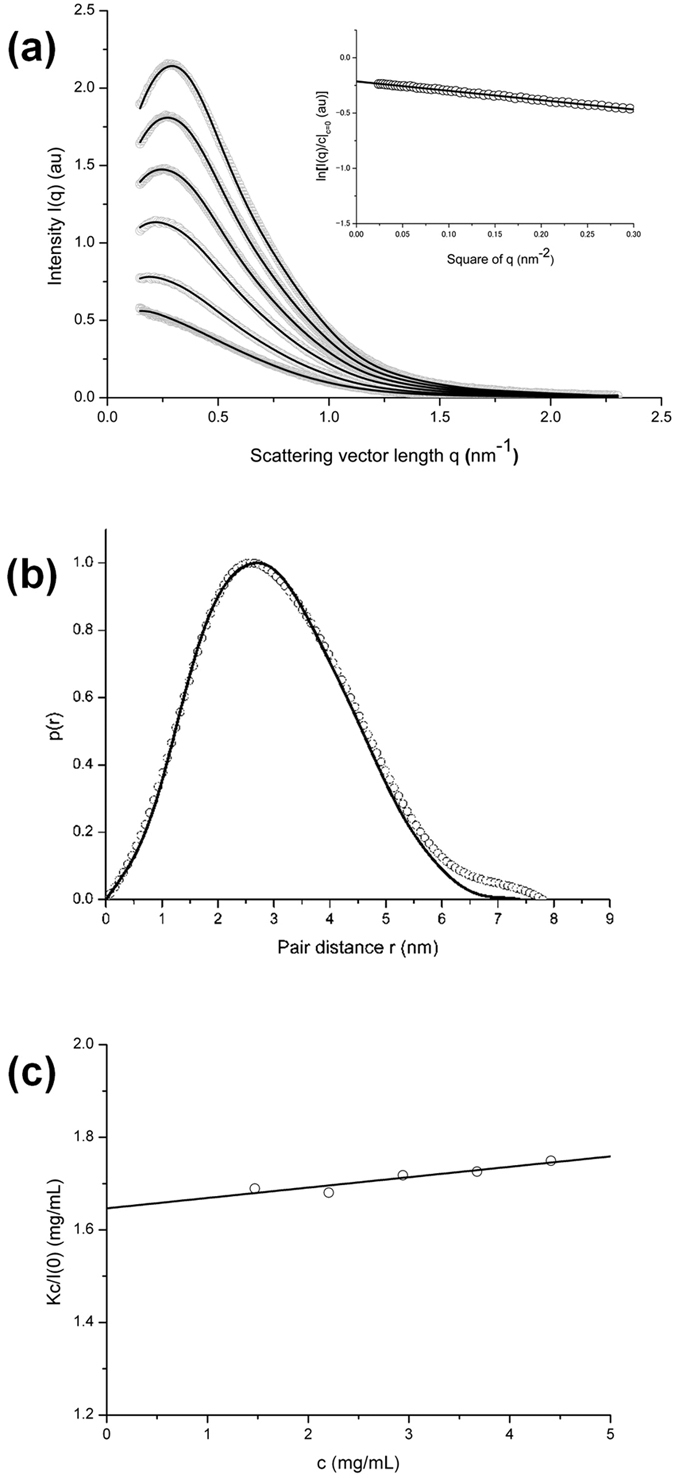
X-ray scattering property of G-actin in G-buffer. (**a)** SAXS intensity profiles from G-actin in G-buffer measured at 10 °C. Open circles are the experimental intensity data measured with actin concentrations (*c*) of 23.3, 34.9, 52.5, 69.8, 87.4 and 105 μM respectively from the bottom in order of increasing concentration. Solid lines are theoretical curves derived from Supplementary Eq. 3 with *r*_*0*_ = 10.6 nm and *β* = 6.084 nm^−1^. Inset shows the Guinier plot of *I*(*q*)/*c|*_*c=0*_ data [ln[*I*(*q*)/*c|*_*c=0*_] versus *q*^2^] and the fitted straight line. Ca-actin was used for the measurements. (**b**) The pair distance distribution function, *p*(*r*). Open circles are the experimental profile, calculated by indirect Fourier transformation of extended *I*(*q*)/*c|*_*c=0*_data. The solid curve was calculated from a crystal structure of actin (PDB code: IJ6Z^25^). *D*_*max*_ is defined as the *r*-intercept of *p*(*r*). (**c**) The *Kc*/*I*(0) *versus c* plot, where *I*(0) is the intensity at *q* = 0, which was derived from the extrapolation of the straight line of the Guinier plot. *Kc*/*I*(0) = 1/*M* + 2*A*_2_*c* + ---, where *K* is the constant, *M* is the molecular weight of G-actin and *A*_*2*_ is the second virial coefficient^22^. A positive value of *A*_*2*_ implies a repulsive interaction between molecules in solution. In this plot, the actin concentration is denoted in mg/ml unit.

Figure 1a shows SAXS intensity profiles from various concentrations of G-actin in G-buffer (low salt solution at pH 8.0, see Methods). We measured SAXS intensities [*I*(*q*), *q*: the scattering vector length] with a protein concentration series ranging from 24 to 107 μM at 10 °C. These profiles exhibit a typical liquid-like behavior that is characterized by a fall-off of scattering intensities at low *q*^21^, due to interference between actin molecules in G-buffer. The magnitude of the fall-off was larger with increasing the concentration of actin (*c*). To empirically correct these inter-particle interference effects, we extrapolated linearly the *I*(*q*)/*c versus q* plots to *c* = 0 at each *q* to obtain *I*(*q*)/*c|*_*c=0*_ data^21^,^22^. From the limiting slope of the linear region of a Guinier plot (see Methods) of *I*(*q*)/*c|*_*c=0*_ data (inset in Fig. 1a), the radius of gyration (*R*_*g*_) of actin was estimated to be 2.41 ± 0.19 nm, a value somewhat smaller than that reported in previous work^23^. From the *y*-intercept of the extrapolated straight line in the Guinier plot [*I*(0)], the molecular weight (*M*) relative to that of bovine serum albumin (as a reference specimen: *M* = 66.5 kDa) was determined to be 41.4 kDa, which is comparable to the known molecular weight of actin molecule (42.3 KDa). Figure 1b shows the pair distance distribution function [*p*(*r*)] that was calculated by indirect Fourier transformation of *I*(*q*)/*c|*_*c=0*_ data^21^,^24^, exhibiting a less symmetric distribution of *r* about a maximum. The *p*(*r*) function was similar to that derived from the crystal structure of G-actin (PDB code: 1J6Z^25^), indicating that G-actin is also an asymmetric molecule in solution. The maximum chord length of the molecule (*D*_*max*_), which is defined as the *r*-intercept of *p*(*r*)^21^, was ~7.8 nm, consistent with the value obtained by Sato *et al*.^26^, but somewhat greater than that derived from the crystal structure of G-actin (~7.4 nm). These results confirmed that the actin molecules were in a monomeric, monodispersed state in G-buffer.

We estimated the closest approach distance between actin molecules (*r*_*0*_) in G-buffer by applying the formula proposed by Zernike and Prins^27^ for the scattering profiles with liquid-like interference between particles (see Supplementary note 1). The concentration series of *I*(*q*) profiles could be fitted well with *r*_*0*_ = 10.6 nm using Supplementary Eq. 3 (Fig. 1a). For simple estimation of the closest surface separation distance between molecules, the effective sphere radius of actin is calculated to be ~3.1 nm (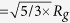) by equating the *R*_*g*_ value of the molecule to a sphere having the same *R*_*g*._ From this value and *r*_*0*_, the surfaces of two actin molecules that closely approach are separated by at least ~4.4 nm. As there is a net negative charge of ~15 electron charges when estimated from amino acid residues of actin, actin molecule (*p*I = 5.4) carries a net negative surface charge at physiological pH, and thus it is screened by the counter-ion layer with a width corresponding to the Debye length (1/*κ* where *κ* is the Debye-Hückel screening parameter^28^,^29^). The Debye length in G-buffer is ~5.1 nm from the ionic strength of solution, comparable to the closest surface separation distance. Thus two actin molecules would repel each other due to the overlap of counter-ion layers^29^ at a separation distance of ~5 nm. Consequently, the actin molecules in G-buffer maintain a monomeric state by an electrostatic repulsion between the molecules, consistent with the observation of a positive slope of the *c*/*I*(0) *versus c* plot (a Zimm plot at *q* = 0)^22^, *i.e.*, the positive value of the second virial coefficient as shown in Fig. 1c.

### Actin solution immediately after mixing with salts

**Figure 2:**
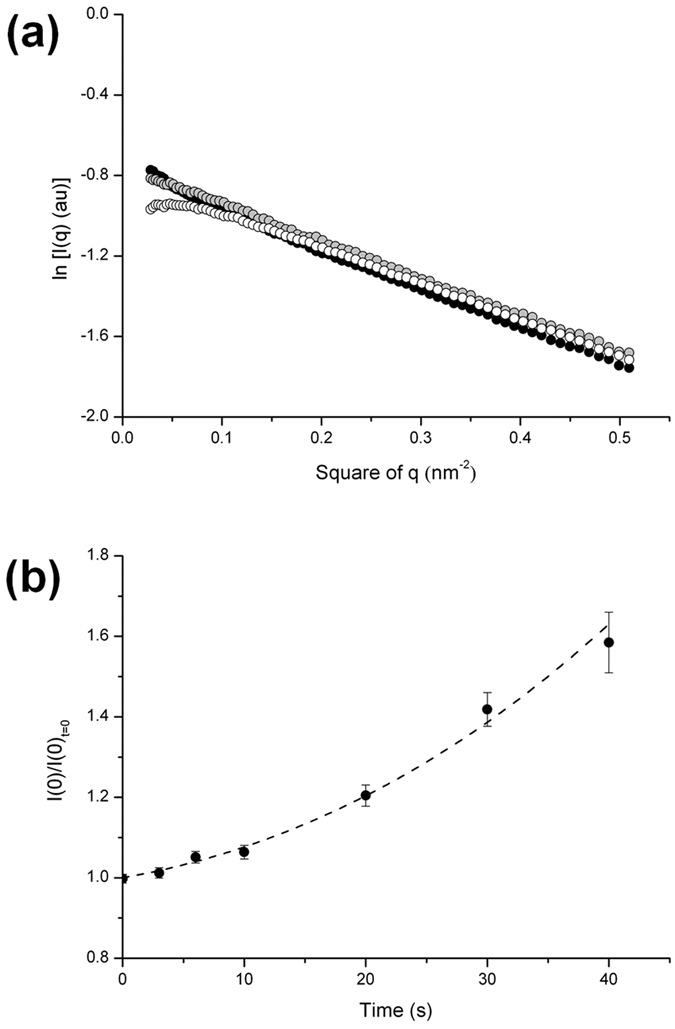
SAXS in the early process of actin polymerization. (**a)** Guinier plots of the SAXS intensity data from an actin solution measured immediately after mixing with salts. Mg-G-actin was mixed at 10 °C with Mg-G-buffer including neutral salts: final concentration, 1 mM MgCl_2_ (gray circles), 100 mM KCl-1 mM MgCl_2_ (black circles) and no additional salts (open circles). Actin concentration after mixing was 31 μM. Each plot is derived from the intensities integrated between 0.010 s and 0.634 s after mixing. The SAXS intensities are corrected by the transmission of each buffer. (**b)** Time courses of the relative zero-angle intensity, *I*(0)_*t*_*/I*(0)_*t=0*_ during polymerization. *I*(0)_*t*_*/I*(0)_*t=0*_ corresponds to the relative weight-averaged molecular weight [*M*_*ave*_(*t*)*/M*]. The final concentration was 100 mM KCl-1 mM MgCl_2_ and the actin concentration after mixing was 31 μM. Each *I*(0) was derived from the *y*-intercept of the extrapolated straight line of a Guinier plot of each *I*(*q*). The theoretical time course of the weight-averaged molecular weight relative to the monomer weight, *M* for the nucleation-controlled polymerization model (for a nucleus size of 4) by Ferrone^17^ (Supplementary Equation (12)) is drawn with the adjustable parameters of α = 2.55 × 10^−5^ s^−2^ and β = 0.098 s^−1^ by the dashed line.

From the situation of G-actin in G-buffer, it is expected that salt-induced polymerization is initiated by shielding of electrostatic repulsive forces between actin molecules and by the reduction of their surface charges *via* salt binding. Figure 2a shows the Guinier plots of SAXS intensity data for 0.5 s after mixing salts, supporting this inference. On mixing equal volumes of G-buffer and G-actin solution of 62 μM, a fall-off of the SAXS intensity at low *q* was observed. The apparent *R*_*g*_ of actin was estimated to be 2.21 ± 0.06 nm from the limiting slope of the linear region in the Guinier plot outside the low-*q* fall-off. In contrast, on mixing equal volume of G-buffer containing 200 mM KCl-2 mM MgCl_2_ and G-actin solution, the inner fall disappeared, though a slight tendency to curve upward at low *q* was occasionally observed. The disappearance of the inner fall-off was very rapid, and any intermediate state was undetected even in the time frame where the scattering intensities were recorded during the period between 0.01 s and 0.06 s after mixing salts. The zero-angle intensity [*I*(0)] was almost identical to that in G-buffer when corrected using electron density of solutes employed. The apparent *R*_*g*_ was 2.43 ± 0.07 nm, close to the true *R*_*g*_ value of 2.41 ± 0.19 nm for G-actin (see inset of Fig. 1a). The *D*_*max*_ value also was comparable to the monomer value. These results indicate that, immediately after mixing salts, the repulsive force between the molecules disappears, while almost all of actin molecules still remain in the monomeric state.

Actin initiates polymerization shortly after the disappearance of the inner intensity fall. Figure 2b shows the time courses of the changes in relative zero-angle intensity [*I*(0)_*t*_/*I*(0)_*t=0*_] early after mixing salts. This corresponds to the time dependence of the weight-averaged molecular weight [*M*(*t*)/*M*(0)]. The curve appeared to be expressed as a cubic function of time with a downward convex shape, ensuring that the actin polymerization under the present conditions is in accordance with a nucleation-controlled reaction scheme^19^ (see Supplementary note 2).

### Time-lapse of SAXS intensity profiles during polymerization of actin

**Figure 3:**
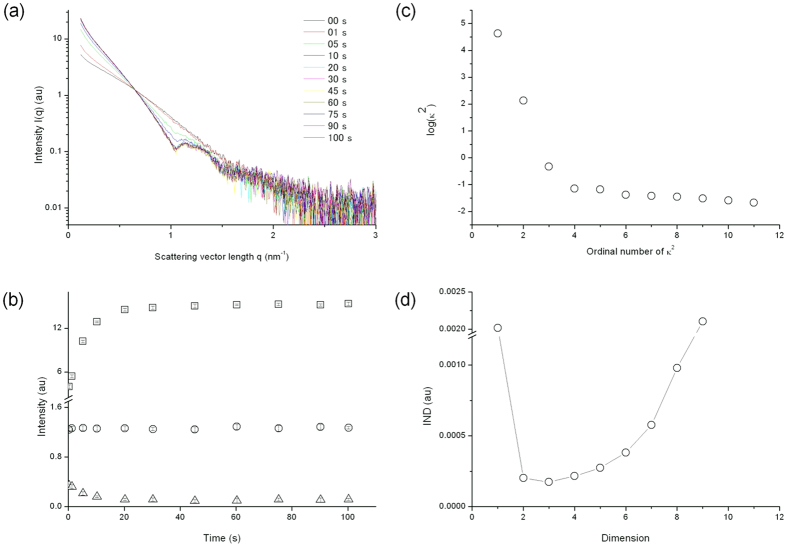
SAXS profiles as a function of time in the whole process of actin polymerization and the singular value and the IND function in singular value decomposition (SVD) analysis. (**a**) A series of SAXS intensity profiles during polymerization of actin measured at 20 °C. The numbers in inset are a time-lapse (*s*) after mixing equal volumes of Mg-G-actin solution of 100 μM and G-buffer including 200 mM KCl and 2 mM MgCl_2_. (**b**) Time-dependent intensity changes during polymerization at *q* = 0.170 (square), 0.647 (circle) and 1.050 nm^−1^ (triangle). The data were taken from (**a)**. (**c**) The singular values (***κ***^**2**^) were obtained by SVD analysis for the set of SAXS intensity data matrix where SAXS intensities were collected row-wise. Log(***κ***^**2**^) values are plotted against the ordinal number (dimension) of (***κ***^**2**^) in decreasing order. (**d**) The plot of the indicator (IND) function values for the empirical determination of the rank of SAXS intensity data matrix. For details, see text.

Figure 3a shows the time-resolved SAXS intensity profiles during salt-induced polymerization of actin. The polymerization was initiated by adding 200 mM KCl and 2 mM MgCl_2_ into the G-actin solution of 100 μM in equal volumes at 20 °C. The SAXS intensity profiles at 20 s or later after mixing showed a clear valley at *q* = 1.05 nm^−1^ and a broad doublet peak between *q* = 1.13 nm^−1^ and *q* = 1.36 nm^−1^. These were characteristics of the SAXS profile for F-actin^23^,^26^, showing the emergence of F-actin.

The time-dependent intensities at *q* = 0.170 nm^−1^ were approximately a mirror-image of those at *q* = 1.05 nm^−1^, as shown in Fig. 3b; both intensity changes appeared to obey simple exponential kinetics and reached a plateau at 20 s after mixing salts. In contrast, the intensity at *q* = 0.647 nm^−1^ was nearly constant, envisaging that this crossing point observed in Fig. 3a is an isoscattering point. This crossing point was also observed in the SAXS profiles measured at 10 °C (Supplementary Figure 1). The existence of the isoscattering point suggests that the polymerization of actin may be described as a two-state transition between G- and F-actin without an intermediate phase. These results were consistent with the prior SAXS results by Matsudaira *et al*.^23^. However, when singular value decomposition (SVD) analysis^30^ (see Methods and Supplemental note 3) was applied to the time-resolved SAXS data, we found that there exist three components that contribute significantly to the scattering intensities. Figures 3c depicts the singular values (*κ*^2^) of SAXS intensity matrix that were obtained. Mallnowski proposed the indicator (IND) function for the determination of the number of factors included in the data from the SVD^31^. Figure 3d shows a plot of the values of IND function derived from the resulting *κ*^2^ against the dimension. The dimension giving the minimum of the IND function is 3. This value exhibits the number of factors sufficient for the description of the SAXS data, corresponding to the minimum number of the kinds of complexes existing in the process of polymerization. Therefore, there is a third complex, other than G- and F-actin, during the polymerization.

### Early stages of salt-induced polymerization of actin by deconvolution analysis of SAXS data

To find the third complex, we examined what oligomers contribute largely to the SAXS intensity profiles during the early stage of polymerization at 10 °C (0 s < *t* ≤ 60 s; see Supplementary Figure 1). There is little prior information on the structure of the oligomers available, but there would be a geometrical restriction imposed by the apparent isoscattering point at *q* = 0.65 nm^−1^ observed in all SAXS profiles. The SAXS profiles are described by scattering terms from individual actin molecules constituting oligomers as well as monomers in solution and interference terms between the actin molecules in oligomers^22^. At such an isoscattering point, the interference terms for all oligomer species (larger than the trimer) should be zero (see Supplementary note 4). This suggests that the oligomers formed during actin polymerization share the same structural periodicity as in the F-actin filament. Therefore it would be sufficient to postulate F-actin-like helical oligomers for scattering analysis of polymerization process. For the following analysis of time-resolved SAXS data, helical oligomers consisting of 3–7 molecules in addition to monomers and dimers were assumed to be involved as the components existing during early polymerization. Two kinds of dimers, those with a parallel configuration (Supplementary Figure 2a) and an anti-parallel configuration (Supplementary Figure 2a), were taken into consideration. Such a dimer with an anti-parallel configuration has frequently been detected at the early stage of polymerization of actin^32^,^33^ despite the fact that this configuration does not exist in normal F-actin. The scattering profiles of these two dimers are distinctive. The scattering intensity profiles calculated for the constructed oligomer models together with these dimer models are depicted in Supplementary Figure 3 and employed as the theoretical intensity profiles.

**Figure 4:**
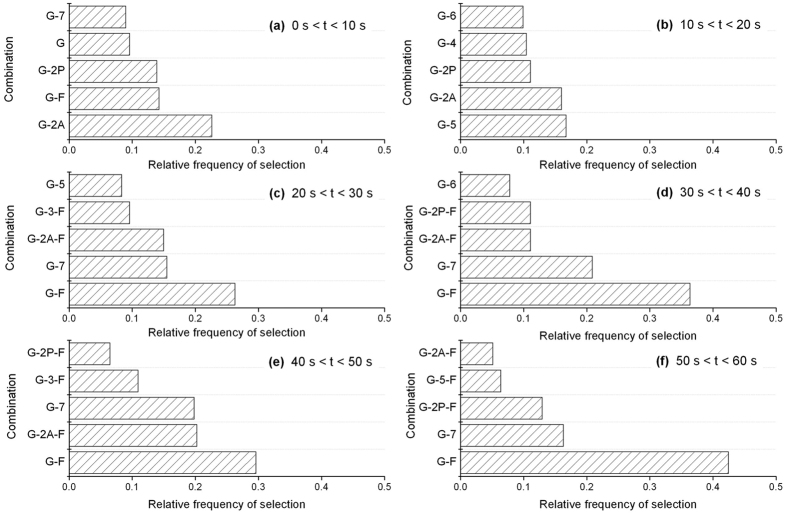
Frequency histograms of the preferred species combination to fit the SAXS intensity profiles within various time divisions during early polymerization. (**a**) 0 s < *t* < 10 s. (**b**) 10 s < *t* < 20 s. (**c**) 20 s < *t* < 30 s. (**d**) 30 s < *t* < 40 s, (**e**) 40 s < *t* < 50 s. (**f**) 50 s < *t* < 60 s. The histograms were made from 16 time-series of the SAXS intensity profiles during polymerization. The reaction was initiated by mixing equal volumes of salt-adding G-buffer and G-actin solutions (final concentration, 100 mM KCl - 1 mM MgCl_2_ and 31 μM actin concentration) at 10 °C. The marks of the species combination express as follows: G (G-actin), 2A (anti-parallel dimer), 2P (parallel dimer), 3 (trimer), 4 (tetramer), 5 (pentamer), 6 (hexamer), 7 (heptamer) and F (F-actin).

To find which combination of the 9 species (G-actin, two kinds of dimers, helical oligomers including 3–7 molecules and F-actin) dominate the SAXS intensity profiles, we utilized the Akaike’s information criterion (AIC)^34^ (see Methods) that has been employed for the selection of a minimum preferred model required for description of data. For all of possible combinations of the species, each time-resolved SAXS intensity profile was fit to a weighted sum of the theoretical profiles of the species, from which the results the AIC were calculated. The requisite minimum combination giving the lowest AIC for each SAXS intensity profile was selected among the 511 (=2^9^ − 1) candidates of combination. Figure 4 shows the frequencies of species combinations that were selected for the intensity profiles in each 10-s time division. In the time division between 0 s and 10 s after mixing salts (Fig. 4a), the combination set of G-actin and anti-parallel dimer was selected as the requisite minimum combination for 22 % of intensity profiles, the frequency of it being much higher than the other sets. Hence, the SAXS intensity profiles at the initial stage between 0 s and 10 s are expressed dominantly by a mixture of G-actin and anti-parallel dimer. At the stages between 20 s and 60 s, the profiles are described predominantly by the set of G-actin and F-actin (Fig. 4c–f). Collectively, the anti-parallel dimer is formed at the initial stage after mixing salts, and F-actin appears thereafter. Therefore, third dominant complex present during early polymerization is the anti-parallel dimers.

**Figure 5:**
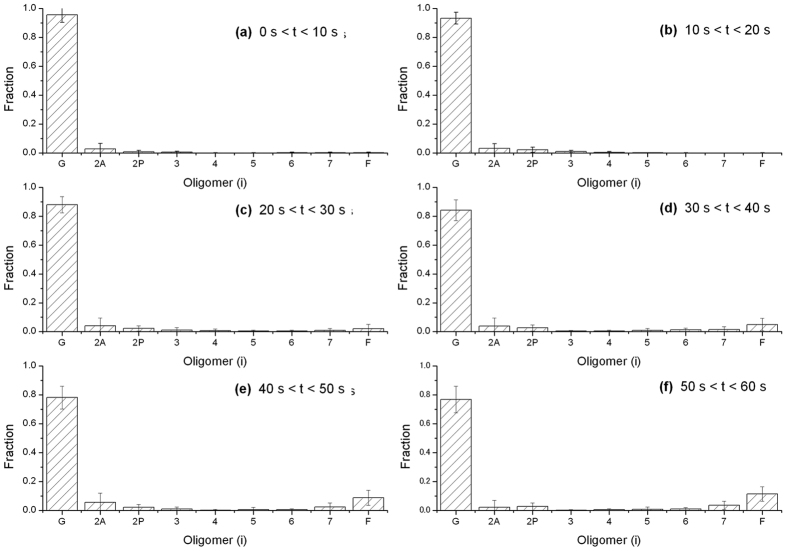
Normalized weight fractions of G-actin, dimers, various oligomers and F-actin derived from deconvolution analysis of SAXS intensity data within various time divisions during early polymerization. (**a**) 0 s < *t* < 10 s. (**b**) 10 s < *t* < 20 s. (**c**) 20 s < *t* < 30 s. (**d**) 30 s < *t* < 40 s. (**e**) 40 s < *t* < 50 s, and (**f**) 50 s < *t* < 60 s. The bars express the weight fractions for oligomer species [G-actin (G), dimers (2A and 2P), trimer (3) to heptamer (7) and F-actin (F)] with error bars of the standard deviations for species. The SAXS data are the same as shown in Fig. 4.

As another example of the weight fractions obtained in the AIC analysis, Fig. 5 shows the weight fractions of each species present in time divisions during early polymerization when the deconvolution was performed using the combination of all species. Characteristics of the weight fractions are consistent with the description deduced from the above minimum preferred model. In addition, the oligomers other than the anti-parallel and parallel dimers, and heptamer do not appreciably accumulate at the early stage of polymerization. The fraction of F-actin increases gradually after a delay of about 20 s. This is supportive evidence that the actin polymerization under the present conditions is a nucleation-controlled reaction^19^.

### X-ray kinetic analysis for polymerization of actin and the free energy change for *i*-mer formation

To investigate the kinetics for polymerization of actin, we analyzed an actin concentration series of the time-resolved SAXS intensity profiles during the early polymerization period of 0 s < t ≤ 60 s, which were measured under the conditions of 100 mM KCl and 1 mM MgCl_2_ at 10 °C and pH 8.0, and estimated the kinetic rate constants and the equilibrium constants in each step of polymerization reaction.

We set up the following kinetic model including the formation of anti-parallel (non-polymerizable) dimers (*A*_*s*_), in which actin molecules (*A*) polymerize in sequence from G- to F-actin^35^,^36^, (see Supplementary note 5).


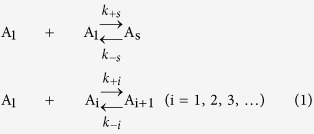


In the above reaction scheme, it is generally assumed that once the oligomer *i* gets to be large, the rate constants for elongation become that for F-actin regardless of the size *i*^16^,^35^. In the present analysis, *k*_+*i*_ = 5.5 μM^−1^s^−1^ and *k*_−*i*_ = 1.8 s^−1^ were assumed for the filament elongation. These values were calculated from the activation enthalpy for polymerization of actin (~6.9 kcal/mol)^37^ and the rate constants at pH 8.3 and room temperature (*k*_+_ = 9.4 μM^−1^s^−1^ and *k*− = 3.1 s^−1^)^38^. These are half of the standard reference values obtained by Pollard under the condition of 50 mM KCl, 1 mM MgCl_2_ and CaCl_2_ in the presence of 0.2 mM ATP (pH 7.0 and 22 °C)^39^. We examined from which step *i* the rate constants can be fixed to *k*_+*i*_ = 5.5 μM^−1^s^−1^ and *k*_−*i*_ = 1.8 s^−1^. First, the reaction model was formulated with the parameter set such that *k*_+*s*_, *k*_−*s*_, *k*_+*i*_ and *k*_−*i*_ (*i* = 1, 2) are variable parameters, and *k*_+*i*_ = 5.5 μM^−1^s^−1^ and *k*_−*i*_ = 1.8 s^−1^ for *i* > 3. With this parameter set, the time-resolved SAXS intensity profiles during the early polymerization in series of 30.9, 40.4 and 49.9 μM actin concentrations were simultaneously fit to those calculated from the models, and the quality of fit between the observed and calculated profiles was described by the χ^2^ -statistics together with a visual inspection. The reduced χ^2^ value was ~1.44 and the free reduced χ^2^ value was ~1.89. Second, *k*_+*3*_ and *k*_−*3*_ were added into the set of variable parameters, resulting that the reduced χ^2^ was decreased to ~1.15 and the free reduced χ^2^ value was ~1.50. Third, further additions of *k*_+*4*_ and *k*_−*4*_, however, did not improve the goodness-of-fit between the observed and calculated profiles. Hence, we adopted the reaction model in which *k*_*s*+_, *k*_*s*−_, *k*_+*i*_ and *k*_−*i*_ (*i* < 3) are variable parameters, and *k*_+*i*_ = 5.5 μM^−1^s^−1^ and *k*_−*i*_ = 1.8 s^−1^ for *i* > 4. The rate constants and the equilibrium constants (*K*_*i*_ = *k*_+*i*_/*k*_−*i*_) obtained here, are given in Supplementary Table 1. It is worth mentioning that the observed data could not be fitted well by the reaction scheme without including the non-polymerizable dimers.

**Figure 6:**
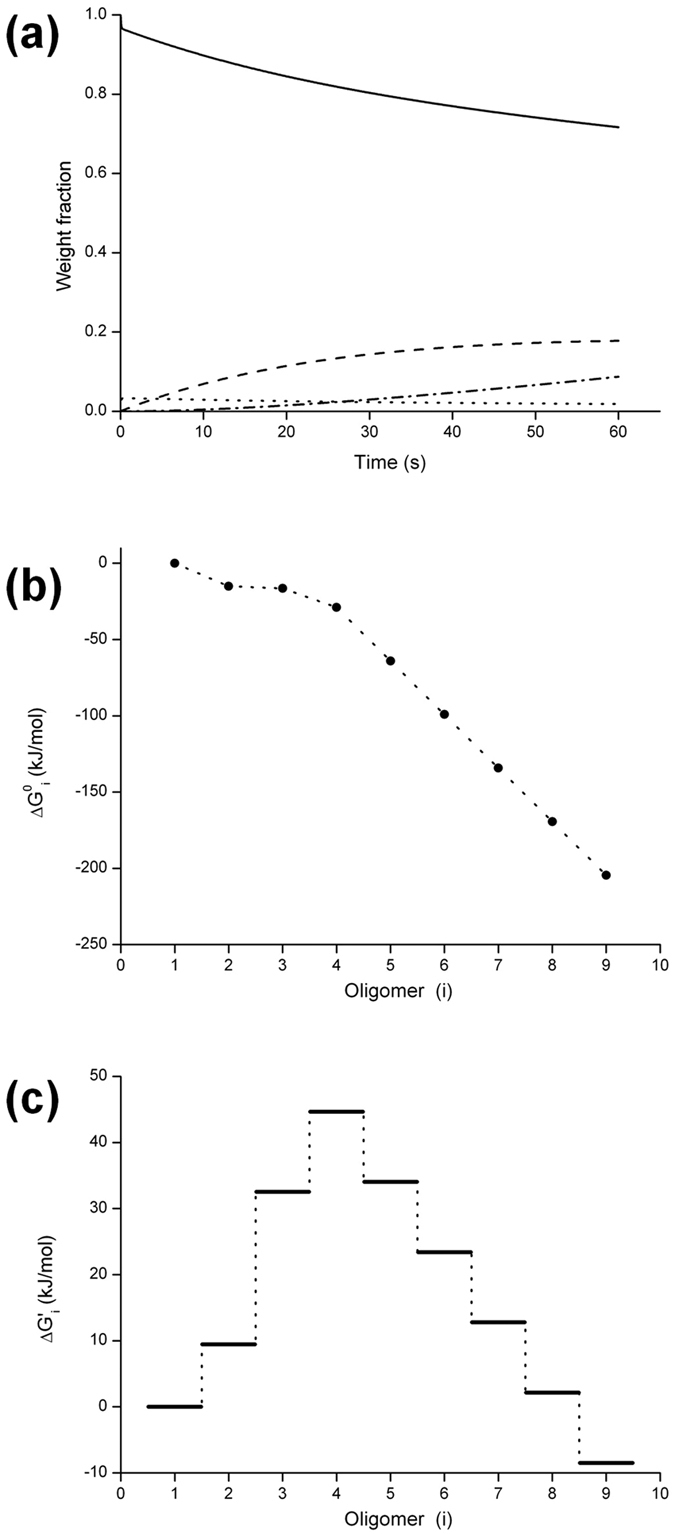
Kinetic analysis of time-resolved SAXS intensities during early polymerization of actin and the free energy changes of *i*-mer formation. (**a**) The time courses of the amounts of G-actin, dimers, oligomers, and F-actin existing during the early polymerization of actin. G-actin (solid), non-polymerizable, anti-parallel dimer (dash), and F-actin (dash-dot) are major components. A small amount of polymerizable, parallel dimer (dot) is also observed. The amounts of the other oligomers are nearly zero. (**b)** Diagram of *ΔG*^*0*^_*i*_ for *i*-mer formation. The diagram corresponds to that of the apparent free energy change (below) in presence of 1M actin. The type of diagram is classified as a cooperative downhill polymerization^20^, and the nucleus is defined as an oligomer (tetramer) at the flexing point of the change of *ΔG*_*i*_^*0*^ versus *i* (d*ΔG*_*i*_^*0*^/d*i*). (**c**) Diagram for *ΔG′*_*i*_ in the presence of 30 μM actin. The type of diagram is a nucleation-controlled polymerization in the category of cooperative polymerization^20^. The nucleus is a tetramer corresponding to a maximum in the free energy diagram.

We derived the time-course of the weight fractions of species existing during the early polymerization together with the fraction of monomers and the result is shown in Fig. 6a. It turned out that the anti-parallel dimer is dominantly formed at the earliest stage just after mixing salts, and F-actin appears thereafter. A small amount of parallel dimer is also observed for the first 60 s. The trends were mostly consistent with the result of the AIC analysis described above. The percentage of non-polymerizable dimer present was estimated to be ~18% of the total actin, and the value was close to 10–15% in previous cross-linking experiments^32^,^33^.

The standard free energy change relative to the monomer upon the formation of *i*-mer, *ΔG*^*0*^_*i*_, was calculated *via* the thermodynamic relation: *ΔG*^*0*^_*i*_ = −*RT* ln(*K*_*1*_*K*_*2*_*…K*_*i−1*_). Figure 6b shows the diagram of *ΔG*^*0*^_*i*_ versus oligomer size (*i*) where the level of monomer is 0. The *ΔG*^*0*^_*2*_ and *ΔG*^*0*^_*4*_ were uniquely determined, but the *ΔG*^*0*^_*3*_ included a small quantity of uncertainty due to the very small fraction of trimer. The diagram indicates that free energy change per monomer addition (the slope of *ΔG*^*0*^_*i*_ versus *i*) alters from the smaller decrement to the larger one at the tetramer (*i* = 4), showing that the oligomer is a transition point from nucleation to elongation. The diagram is compared with those in the previous studies^16^,^18^,^36^,^40^ in Supplementary Figure 4.

To describe the behavior of early polymerization under the present conditions, we defined the apparent free energy change in the presence of constant actin [A]: *ΔG*^*0′*^_*i*_ = *ΔG*^*0*^_*i*_ – *RT*ln([A]^*i−1*^). A diagram of *ΔG*^*0′*^_*i*_ in the presence of 30 μM actin is shown in Fig. 6c. The diagram indicates that the polymerization of actin changes from the energy-requiring reaction (*ΔG*^*0′*^_*i*_ > 0) to the spontaneous reaction (*ΔG*^*0′*^_*i*_ < 0) at the tetramer (*i* = 4). The reactions before the tetramer formation correspond to an unfavorable nucleation while those after the tetramer formation to a favorable elongation. This type of polymerization has been classified as a nucleated cooperative polymerization^20^, where the oligomer with the highest free energy change is a nucleus. Figure 6c shows that it is the tetramer. Steady elongation starts at the pentamer with the same rate constants for monomer addition to F-actin. Thus the pentamer serves as ‘seeds’ for the canonical elongation of actin.

## Discussion

The time-resolved SAXS intensities during salt-induced polymerization of actin appear to have the apparent isoscattering point (Fig. 3a) and to show single exponential kinetics (Fig. 3b). From the similar SAXS observations, Matsudaira *et al*.^23^ asserted that actin molecules condense into filaments without the formation of nuclei; their SAXS analysis showed extensive dimerization of actin and suggested rapid incorporation of the dimers into oligomers. However, the diagram of the free energy change of oligomer formation (Fig. 6c) together with the time-dependence of the average molecular weight during polymerization (Fig. 2b) indicates that the K^+^/Mg^2+^-induced polymerization of actin follows a nucleation-controlled reaction scheme (*i.e.* nucleation is an energetically unfavorable process). According to the definition of Ferrone^19^,^20^, the nucleus of the reaction is a helical tetramer because it is the least stable intermediate oligomer with respect to the monomer and exists only transiently in the polymerization process (Fig. 6c). This conclusion is consistent with the previous, kinetic studies of the actin polymerization by Tobacman and Korn^15^ and by Fesce *et al*.^41^. In parallel to the nucleation, non-polymerizable dimers are formed accumulated during the early polymerization (Figs 4 and 6a). This has been reported by many previous studies though the percentage of the dimer fraction is variable: ~18% of the total actin in this work is consistent with 10–15% derived by cross-linking experiments^32^,^33^, while ~60% in the previous SAXS experiment^23^ (though a distinction of two types of dimers was not made) and ~25% in the small-angle neutron scattering experiment^42^.

The size of the nucleus in actin polymerization has been discussed in terms of the simple geometrical explanations^43^,^44^. As discussed in Supplementary note 6, the formation of a dimer or a trimer as a nucleus has been deduced from the structural geometry of F-actin^36^. However, we showed here that the nucleus for polymerization of actin is a tetramer. The nucleus size is probably related to the structure of the end receiving a monomer. As shown in Fig. 6b, the decrement of standard free energy change *ΔG*^0^ upon the accretion of an additional monomer to the trimer is smaller than that upon the canonical growth. The implication for this is that end of the trimer has an incomplete structure (see Supplementary Figure 5c). By contrast, in Fig. 6b, the decrement of free energy change *ΔG*^0^ upon the addition of a monomer to the tetramer is larger than the case to the trimer, being the same as that on the canonical elongation of filament. This suggests that the structure of the end receiving an additional monomer in the tetramer would be identical to that of the fast growing B-end of F-actin, probably having the same subunit disposition as in F-actin. The subunit disposition would require the flattening of actin molecule that was found upon the association of G-actin to F-actin filament^45^. This is because the flattening is considered to be necessary for generating the canonical subunit arrangements in F-actin^46^. The flattening is produced by a relative rotation of two main domains constituting an actin molecule around the line passing through subdomains 1 and 3, being stabilized by forming the longitudinal contact with the other subunit at subdomains 2 and 4^45^ (see Supplementary Figure 5b). We deduce that two subunits at the B-end of tetramer, colored by red in Supplementary Figure 5c, have a flat conformation, and that the diagonal contacts between the two subunits can generate the canonical disposition like the B-end of F-actin, serving as a nucleus for further polymerization. More detailed discussion based upon crystal structures of actin oligomers will be presented elsewhere.

**Figure 7:**
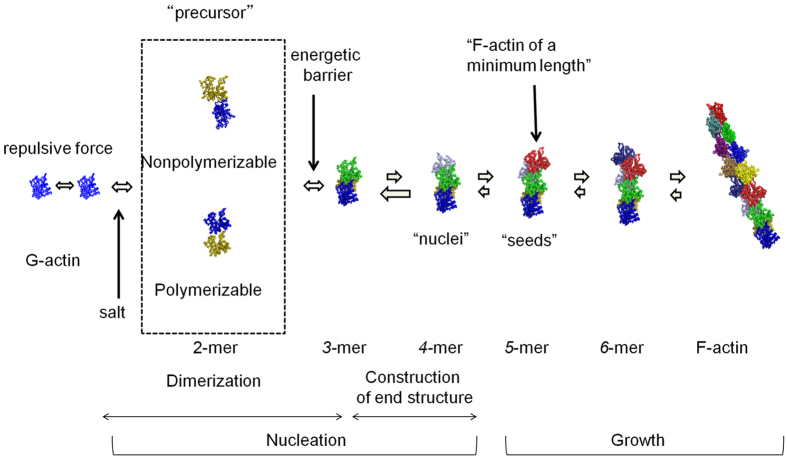
Schematic drawing of the plausible pathway for actin polymerization under the present conditions. Nucleation process includes the formation of dimers, trimer, and tetramer, and followed by canonical growth process is from the tetramer.

In Fig. 7, we summarize our view of the pathway for salt-induced polymerization of actin at the present conditions. The addition of neutral salts diminishes the electrostatic repulsion force between G-actin molecules in G-buffer, allowing them to closely approach each other. Activation of monomer *via* salt binding which is thought to be prerequisite for initiation of polymerization^40^,^43^,^47^ would simultaneously occur, though it could not be detected by the SAXS at the present resolution. The addition of salts engenders a large excess of monomers capable of polymerization through a decrease in the critical concentration (the concentration of monomer in equilibrium with polymer) of actin for polymerization, typically from ca. 109 μM in G-buffer to 0.3 μM in salt solution. Then the dimeric molecules, such as parallel and anti-parallel dimers, are formed prior to a major nucleation. Dimers with an anti-parallel orientation of subunits do not contribute to the nucleation, though they are thought to be incorporated transiently into growing filaments^32^,^33^. Meanwhile, from the polymerizable dimers, the trimer and the tetramer are sequentially formed, though these are energetically unfavorable reactions. Once monomers are added to the tetramers, the reaction proceeds along the free energy downhill slope, and F-actin filaments start to grow. Although the formation of nuclei ceases with a decrease in polymerizable monomers, the growth of filaments continues until the concentration of monomers comes to reach the critical concentration for polymerization, resulting in the accumulation of long F-actin filaments. Non-polymerizable dimers are accumulated but almost disappear at steady state because the binding constant is much higher than the critical concentration for polymerization (see *K*^*−1*^ = 1.6 × 10^2^ μM in Supplementary Table 1).

For rapid generation of new filaments in cells, it would be essential to generate the end structure with a diagonal disposition of subunits similar to the B-end of F-actin and to keep the disposition until the subsequent monomers are added. Two actin molecules help the other two molecules to form the diagonal disposition probably *via* the promotion of flattening in the case of actin alone. By contrast, in cells, nucleators having two or more binding sites to actin molecules, promote a formation of such a diagonal disposition. Disposition of two Arp subunits in the NPF-Arp2/3 complex resembles successive “short pitch helices” in F-actin^48^, to which the subsequent monomer is delivered by the VCA domain of WASp^49^. Formin dimer holds two actin molecules with a face-to-face arrangement that corresponds to a diagonal disposition of successive “short pitch helices”^50^, to which two subsequent monomers are recruited by each DAD motif domain of formin dimer^51^. Cordon-bleu, Spire, Vop-L and JMY align actin molecules to have the longitudinal disposition of subunits as in F-actin by the use of multiple WH2 domains^52^. The longitudinal contact might maintain the actin subunit in the flat conformation, promoting the formation of nucleus. However, the recruitment and delivery of the subsequent monomer are still left behind. Recently, an interesting model for Vop-L has been proposed by Zahm *et al*.^53^; the VCD domains of Vop-L dimer can bind to three successive actin molecules as in F-actin, to which the WH2 domains of Vop-L recruit the next monomers.

## Methods

### Preparation of actin

Actin was prepared from chicken breast muscle according to the method described previously^54^. The chicken breast muscles were obtained from the butcher shop with slaughterhouse (Shibata’s Shyoten, Tatsuno). Actin was furthermore purified by size-exclusion chromatography with a HiLoad Superdex-200 column (GE healthcare). After elution, actin was concentrated by a viva-spin (Sartorius) to about 5 mg/ml, and dialyzed against G-buffer (5 mM Tris-HCl, 0.02 mM CaCl_2_, 0.2 mM ATP, 1 mM NaN_3_ and 0.5 mM DTT, pH 8.0). The resulting G-actin solution was centrifuged at typically 5 × 10^4 ^rpm for 30 min before use. The actin thus obtained was used as Ca-actin. Mg-actin was converted from Ca-actin, typically by addition of 1/10 volume of 2 mM EGTA and 0.8 mM MgCl_2_, and incubated on ice for 5 min and then at 10 °C for 10–15 min each time before measuring SAXS.

### SAXS measurements

SAXS measurements were performed with 3rd generation synchrotron radiation at the 45XU-SAXS beamline of SPring-8, Hyogo, Japan 55. The specimen-to-detector distance was typically 1498 mm. The maximum small-angle resolution of our set-up is nominally 2π/*q*_*min*_ ~ 50 nm, where *q*_*min*_ is the minimum accessible scattering vector length. The SAXS intensity patterns were recorded by the use of a PILATUS 300-k-w photon counting two-dimensional (2D) detector (DECTRIS, Barden, Switzerland). The 2D-intensity patterns were circularly averaged to convert to one-dimensional (1D) intensity profiles using the FIT2D program (by ESRF, Grenoble). The 1D-intensity profiles after averaging for the buffer solution measured under the same experimental conditions were subtracted from those for the actin solution. The net SAXS intensity profiles, *I*(*q*)s were obtained, where *q* is the scattering vector length [= 4πsin*θ*/*λ*, *θ* is a half the scattering angle and *λ*, the wavelength of X-rays (0. 10 nm) used]. A concentration series of the SAXS intensity profiles for actin or bovine serum albumin (BSA, Sigma Aldrich) was measured with a protein concentration (*c*), ranging from 2 to 6 mg/ml, and each profile was extrapolated to *c* = 0. The radius of gyration (*R*_*g*_) of the molecule (the root-mean-square distances of the electrons from the center of gravity of the molecule) and the scattering intensity at zero angle (*q* = 0), *I*(0) were calculated, respectively, from the limiting slope and the *y*-intercept of the extrapolated linear region of the Guinier plot [ln[*I*(*q*)] *versus q*^*2*^] in the range of *qR*_*g*_ < 1.0 (Guinier’s criterion^22^) using PRIMUS^56^. The molecular weight (*M*) was determined from the *I*(0)/*c* relatively to that from BSA (*M* = 66.5 kDa) as a standard specimen. The pair distance distribution function [*p*(*r*)] was calculated by indirect Fourier transformation of extended *I*(*q*)/*c|*_*c=0*_data with the use of GNOM^22^. [*p*(*r*) denotes the frequency of pair-vector lengths, *r* between any two small volume elements within the molecule.] The *r*-intercept yields the maximum chord length (*D*_*max*_) of the molecule.

Static SAXS measurements were performed using the 30 μl-volume X-ray cell provided by the beamline maintaining 10 °C with a Peltier device. Time-resolved SAXS measurements were performed at the same beamline using an improved stopped-flow mixer (the dead time of mixing < 3 ms) (UNISOKU Co., Osaka, Japan) at 10 °C or 20 °C. The apparatus can rapidly mix 100 μl-volumes of two kinds of solutions: in this case, G-actin solution and salt solution. The operation of the stopped flow mixer was synchronized with the data-acquisition system. The measurement sequences were: rapid mixing, opening of the X-ray shutter after a variable time delay, followed by the recording of 10–20 SAXS patterns with an exposure time of 50 ms at intervals of 3 ms at 10 ms after the opening signal of the shutter. The measurement was repeated with a different time delay to obtain data series reflecting the time sequence of polymerization. After checking the lack of any sample damage effects by the X-ray exposure for each pattern, a series of the intensity profiles with the same time delay were averaged to obtain one profile. The average of the time when the recording of each pattern in the series was initiated after mixing [the time delay + 10 ms + (the number of espouse − 1) × (50 + 3) ms] was assigned to the averaged profile as the time-course of polymerization.

### SVD analysis

Time-dependent SAXS intensity data are collected row-wise in the matrix and the SAXS intensity matrix was made. The singular values (*κ*^2^) of the matrix were calculated by the use of the *sdv* command in the Scilab Package^57^. To empirically decide the minimum number of oligomeric components, we calculated the indicator (IND) function that was proposed by Mallnowski^30^. The number (dimension) giving the lowest value of the IND function is the substantial rank of the matrix (see Supplementary note 3).

### Deconvolution of the SAXS intensity profiles

The weight fractions of G-actin, dimers, various oligomers and F-actin during polymerization at time *t* were estimated by the minimization of the following function using a quadratic programming problem solver in the Scilab Package^57^ under the conditions of *f*_*i*_(*t*) ≥ 0 and Σ_*i*_ *f*_*i*_ (*t*) = 1,


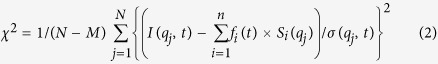


where *I*(*q*_*j*_, *t*) is the SAXS intensity measured at *q*_*j*_ with the standard deviation of errors [*σ*(*q*_*j*_, *t*)] at time *t*, *N* is the number of points in the intensity profiles, *M* is a number of freedom in the fitting, *f*_*i*_*(t)* is the weight fraction of species *i*, and *S*_*i*_(*q*_*j*_) is the corresponding SAXS intensity at *q*_*j*_ from each model for species *i* which represents G-actin, various intermediates and F-actin. Scattering profiles for the oligomer models were calculated with CRYSOL^58^ from atomic coordinates. Coordinates for the anti-parallel dimer were made from the arrangement of the anti-parallel dimer in the actin crystal of PDB code: 1LCU^59^, and those for the parallel dimer were made from the arrangement along the crystal contacts of PDB code: 2FXU^60^. Coordinates for F-actin-like helical oligomers were prepared from the F-actin model by Oda *et al*. (PDB code: 2ZWH)^45^. The model scattering profile of G-actin was based on the SAXS intensity data extrapolated to c = 0 and the SAXS intensity data immediately after mixing salt. That of F-actin was based on the SAXS intensity data in the fully-polymerized state at 100 mM KCl and 1 mM MgCl_2_. Scaling of the scattering profiles from these models other than the dimers and the trimer was done using the scattered intensities at the apparent isoscattering point at *q* = 0.65 nm^−1^. The model intensity profiles of the dimers and the trimer were scaled against that of the tetramer using their *I*(0) values.

### AIC Analysis

We selected randomly halves of the intensity data that were included in the 10–20 SAXS profiles recorded successively for an exposure time of 50 ms at intervals of 3 ms with a time delay after mixing salts. We calculated the averages and their deviations of intensities from the selected profiles at each scattering vector length *q*. One hundred averaged profiles with the deviations were prepared from one measurement. We treated one hundred SAXS intensity data not as one averaged data to reduce the effect of random errors included in each measurement on deconvolution. These intensity profiles were deconvoluted by all sets of possible combinations of the model profiles obtained from G-actin, anti-parallel dimers, parallel dimers, trimers, tetramers, pentamers, hexamers, heptamers and F-actin. The AIC^34^ was calculated for each combination and each profile, and AIC is defined as


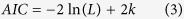


where *L* is the maximum value of a likelihood function and *k* is the number of parameters used in the model. We adopted the combination of the species giving the lowest AIC value as a preferred combination for each averaged profile. The procedure was performed for 16 independent time-series of the SAXS intensity profiles that were measured under the same experimental conditions.

### X-ray kinetic analysis using the sequential polymerization model

Plausible rate constants were determined by fitting the SAXS intensity profiles that were computed using the kinetic scheme of polymerization (see Supplementary note 5) to the observed time-resolved SAXS intensity profiles with actin concentrations of 30.9, 40.4 and 49.9 μM during the early polymerization period (0 s < t ≤ 60 s). The fitting procedure was performed through the minimization of the reduced χ^2^ for the SAXS profiles in each time series using *fminsearch* command in the Scilab Package^57^. The free reduced χ^2^ was also calculated for the data points that were selected randomly at the ratio of one per twenty points in low *q* range. The fitting was performed by the set including both the variable parameters, *k*_+*s*_, *k*_−*s*_, *k*_+*i*_ and *k*_−*i*_ (*i* < *i*_*0*_), and the fixed parameters, *k*_+*i*_ = 5.5 μM^−1^s^−1^ and *k*_−*i*_ = 1.8 s^−1^ (*i* ≥ *i*_*0*_) where *i*_*0*_is a minimum length of the canonical elongation. The constraint condition, *k*_+*i*_ < 5.5 μM^−1^s^−1^, was added for the fitting. First, *i*_*0*_ giving the minimum values of the reduced χ^2^ was decided by fitting with changing *i*_*0*_. Second, under the condition of the minimal *i*_*0*_, the initial sets of parameters were systematically, randomly generated, from which the plausible sets of the rate constants were searched for. We prepared 168 initial sets.

## Additional Information

**How to cite this article**: Oda, T. *et al*. Early nucleation events in the polymerization of actin, probed by time-resolved small-angle x-ray scattering. *Sci. Rep.* **6**, 34539; doi: 10.1038/srep34539 (2016).

## Supplementary Material

Supplementary Information

## References

[b1] Carlier M. F. & Pantaloni D. Control of actin assembly dynamics in cell motility. J. Biol. Chem. 282, 23005–23009 (2007). 17576764 10.1074/jbc.R700020200

[b2] Pollard T. D. Regulation of actin filament assembly by Arp2/3 complex and formins. Annu. Rev. Biophys. Biomol. Struct. 36, 451–477 (2007). 17477841 10.1146/annurev.biophys.35.040405.101936

[b3] Mullins R. D., Heuser J. A. & Pollard T. D. The interaction of Arp2/3 complex with actin: nucleation, high affinity pointed end capping, and formation of branching networks of filaments. Proc. Natl. Acad. Sci. USA 95, 6181–6186 (1998). 9600938 10.1073/pnas.95.11.6181 PMC27619

[b4] Pruyne D. . Role of formins in actin assembly: nucleation and barbed-end association. Science 297, 612–615 (2002). 12052901 10.1126/science.1072309

[b5] Graziano B. R. . Mechanism and cellular function of Bud6 as an actin nucleation-promoting factor. Mol. Biol. Cell 22, 4016–4028 (2011). 21880892 10.1091/mbc.E11-05-0404 PMC3204064

[b6] Quinlan M. E., Heuser J. E., Kerkhoff E. & Mullins R. D. Drosophila Spire is an actin nucleation factor. Nature 433, 382–388 (2005). 15674283 10.1038/nature03241

[b7] Ahuja R. . Cordon-bleu is an actin nucleation factor and controls neuronal morphology. Cell 131, 337–350 (2007). 17956734 10.1016/j.cell.2007.08.030 PMC2507594

[b8] Chereau D. . Leiomodin is an actin filament nucleator in muscle cells. Science 320, 239–243 (2008). 18403713 10.1126/science.1155313 PMC2845909

[b9] Zuchero J. B., Coutts A. S., Quinlan M. E., Thangue N. B. & Mullins R. D. p53-cofactor JMY is a multifunctional actin nucleation factor. Nat. Cell. Biol. 11, 451–459 (2009). 19287377 10.1038/ncb1852 PMC2763628

[b10] Okada K. . Adenomatous polyposis coli protein nucleates actin assembly and synergizes with the formin mDia1. J. Cell Biol. 189, 1087–1096 (2010). 20566685 10.1083/jcb.201001016 PMC2894447

[b11] Dominguez R. Structural insights into de novo actin polymerization. Curr. Opin. Struct. Biol. 20, 217–225 (2010). 20096561 10.1016/j.sbi.2009.12.012 PMC2854303

[b12] Straub F. B. Actin. Studies Int. Med. Chem. Univ. Szeged 2, 3–15 (1942).

[b13] Oosawa F. & Kasai M. A theory of linear and helical aggregations of macromolecules. J. Mol. Biol. 4, 10–21 (1962). 14482095 10.1016/s0022-2836(62)80112-0

[b14] Wegner A. & Engel J. Kinetics of the cooperative association of actin to actin filaments. Biophys. Chem. 3, 215–225 (1975). 1174645 10.1016/0301-4622(75)80013-5

[b15] Tobacman L. S. & Korn E. D. The kinetics of actin nucleation and polymerization. J. Biol. Chem. 258, 3207–3214 (1983). 6826559

[b16] Frieden C. Polymerization of actin: mechanism of the Mg^2+^-induced process at pH 8 and 20 °C. Proc. Natl. Acad. Sci. USA 80, 6513–6517 (1983). 6579538 10.1073/pnas.80.21.6513 PMC390383

[b17] Fujiwara I., Vavvlonis D. & Pollard T. D. Polymerization kinetics of ADP- and ATP-Pi-actin determined by fluorescence microscopy. Proc. Natl. Acad. Sci. USA 104, 8827–8832 (2007). 17517656 10.1073/pnas.0702510104 PMC1885587

[b18] Husson C., Renault L., Didry D., Pantaloni D. & Carlier M. F. Cordon-bleu uses WH2 domains as multifunctional dynamizers of actin filament assembly. Mol. Cell 43, 464–477 (2011). 21816349 10.1016/j.molcel.2011.07.010

[b19] Ferrone F. Analysis of protein aggregation kinetics. Methods Enzymol. 309, 256–274 (1999). 10507029 10.1016/s0076-6879(99)09019-9

[b20] De Greef T. F. A. . Supramolecular polymerization. Chem. Rev. 109, 5687–5754 (2009). 19769364 10.1021/cr900181u

[b21] Pilz I. Proteins in Small Angle X-ray Scattering (eds Glatter O. & Kratky O.) Ch. 8, 239–293 (Academic Press, 1982).

[b22] Porod G. General Theory in Small Angle X-ray Scattering (eds Glatter O. & Kratky O.) Ch. 2, 17–51 (Academic Press, 1982).

[b23] Matsudaira P., Bordas J. & Koch M. H. J. Synchrotron X-ray diffraction studies of actin structure during polymerization. Proc. Natl. Acad. Sci. USA 84, 3151–3155 (1987). 3472201 10.1073/pnas.84.10.3151 PMC304826

[b24] Svergun D. I. Determination of the regularization parameter in indirect-transform methods using perceptual criteria. J. Appl. Cryst. 25, 495–503 (1992).

[b25] Otterbein L. R., Graceffa P. & Dominguez R. The crystal structure of uncomplexed actin in the ADP state. Science 293, 708–711 (2001). 11474115 10.1126/science.1059700

[b26] Sato T. . Actin oligomers at the initial stage of polymerization induced by increasing temperature at low ionic strength: Study with small-angle X-ray scattering. BIOPHYSICS 6, 1–11 (2010). 10.2142/biophysics.6.1 PMC5036667 27857581

[b27] Zernike F. & Prins J. A. Die Beugung von Röntgenstrahlen in Flüssigkeiten als Effekt der Molekülanordnung Z. Phys. 41, 184–194 (1927).

[b28] Israelachvilli J. N. Intermolecular and Surface Forces with Applications to Colloidal and Biological Systems (Academic Press, 1985).

[b29] Ohshima H. Theory of colloid and interfacial electric phenomena. (Elsevier B.V., 2006).

[b30] Mallnowski E. R. Determination of the number of factors and the experimental error in a data matrix. Anal. Chem. 49, 612–617 (1977).

[b31] Mallnowski E. R. Theory of error in factor analysis. Anal. Chem. 49, 606–612 (1977).

[b32] Galinska-Rakoczy A., Wawro B. & Strzelecka-Golaszewska H. New aspects of the spontaneous polymerization of actin in the presence of salts. J. Mol. Biol. 387, 869–882 (2009). 19340945 10.1016/j.jmb.2009.02.011

[b33] Grintsevich E. E. . Antiparallel dimer and actin assembly. Biochemistry 49, 3919–3927 (2010). 20361759 10.1021/bi1002663 PMC3133779

[b34] Akaike H. A new look at the statistical model identification. IEEE Trans. Automat. Contr. 19, 716–723 (1973).

[b35] Frieden C. & Goddette D. W. Polymerization of actin and actin-like systems: evaluation of the time course of polymerization in relation to the mechanism. Biochemistry 22, 5836–5843 (1983). 6661414 10.1021/bi00294a023

[b36] Sept D. & McCammon J. A. Thermodynamics and kinetics of actin filament nucleation. Biophys. J. 81, 667–674 (2001). 11463615 10.1016/S0006-3495(01)75731-1 PMC1301543

[b37] Kinosian H. J., Selden L. A., Estes J. E. & Gershman L. C. Thermodynamics of actin polymerization; influence of the tightly bound divalent cation and nucleotide. Biochim. Biophys. Acta 1077, 151–158 (1991). 2015289 10.1016/0167-4838(91)90052-2

[b38] Crevenna A. H. . Electrostatics control actin filament nucleation and elongation kinetics. J. Biol. Chem. 288, 12102–12113 (2013). 23486468 10.1074/jbc.M113.456327 PMC3636895

[b39] Pollard T. D. Rate constants for the reactions of ATP- and ADP-actin with the ends of actin filaments. J. Cell Biol. 103, 2747–2754 (1986). 3793756 10.1083/jcb.103.6.2747 PMC2114620

[b40] Cooper J. A., Buhle E. L. Jr., Walker S. B., Tsong T. Y. & Pollard T. D. Kinetic evidence for a monomer activation step in actin polymerization. Biochemistry 22, 2193–2202 (1983). 6860660 10.1021/bi00278a021

[b41] Fesce R., Benfenati F., Greengard P. & Valtorta F. Effects of the neuronal phosphoprotein synapsin I on actin polymerization. II. Analytical interpretation of kinetic curves. J. Biol. Chem. 267, 11289–11299 (1992). 1597463

[b42] Goddette D. W., Uberbacher E. C., Bunick G. J. & Frieden C. Formation of actin dimers as studied by small angle neutron scattering. J. Biol. Chem. 261, 2605–2609 (1986). 3949737

[b43] Frieden C. Actin and tubulin polymerization: the use of kinetic methods to determine mechanism. Annu. Rev. Biophys. Biophys. Chem. 14, 189–210 (1985). 3890879 10.1146/annurev.bb.14.060185.001201

[b44] Oosawa F. & Asakura S. Thermodynamics of the Polymerization of Protein. (Academic Press, 1975).

[b45] Oda T., Iwasa M., Aihara T., Maeda Y. & Narita A. The nature of the globular- to fibrous-actin transition. Nature 457, 441–445 (2009). 19158791 10.1038/nature07685

[b46] Oda T. & Maeda Y. Multiple Conformations of F-actin. Structure 18, 761–767 (2010). 20637412 10.1016/j.str.2010.05.009

[b47] Gershman L. C., Newman J., Selden L. A. & Estes J. E. Bound-cation exchange affects the lag phase in actin polymerization. Biochemistry 23, 2199–2203 (1984). 6428448 10.1021/bi00305a015

[b48] Volkmann N., Page C., Li R. & Hanein D. Three-dimensional reconstructions of actin filaments capped by Arp2/3 complex. Eur. J. Cell Biol. 93, 179–183 (2014). 24552843 10.1016/j.ejcb.2014.01.003 PMC4110117

[b49] Ti S. C., Jurgenson C. T., Nolen B. J. & Pollard T. D. Structural and biochemical characterization of two binding sites for nucleation-promoting factor WASp-VCA on Arp2/3 complex. Proc. Natl. Acad. Sci. USA 108, E463–E471 (2011). 21676862 10.1073/pnas.1100125108 PMC3158158

[b50] Otomo T. . Structural basis of actin filament nucleation and processive capping by a formin homology 2 domain. Nature 433, 488–494 (2005). 15635372 10.1038/nature03251

[b51] Gould C. J. . The formin DAD domain plays dual roles in autoinhibition and actin nucleation. Curr. Biol. 21, 384–390 (2011). 21333540 10.1016/j.cub.2011.01.047 PMC3058777

[b52] Carlier M. F., Husson C., Renault L. & Didry D. Control of actin assembly by the WH2 domains and their multifunctional tandem repeats in Spire and Cordon-bleu. Int. Rev. Cell Mol. Biol. 290, 55–85 (2011). 21875562 10.1016/B978-0-12-386037-8.00005-3

[b53] Zaham J. A. . Structure of filament-like actin trimer bound to the bacterial effector Vop-L. Cell 155, 423–434 (2013). 24120140 10.1016/j.cell.2013.09.019 PMC4048032

[b54] Spudich J. A. & Watt S. The regulation of rabbit skeletal muscle contraction. I. Biochemical studies of the interaction of the tropomyosin-troponin complex with actin and the proteolytic fragments of myosin. J. Biol. Chem. 246, 4866–4871 (1971). 4254541

[b55] Fujisawa T. . Small-angle X-ray scattering station at the SPring-8 RIKEN beamline. J. Appl. Cryst. 33, 797–800 (2000).

[b56] Konarev P. V., Volkov V. V., Sokolova A. V., Kochb M. H. J. & Svergun D. I. PRIMUS: A windows PC-based system for small-angle scattering data analysis. J. Appl. Cryst. 36, 1277–1282 (2003).

[b57] Scilab: Free and open source software for numerical computation (OS, Version 5.22) [Software]. Available from: http://www.scilab.org (2012).

[b58] Svergun D. I., Barberato C. & Koch M. H. J. CRYSOL - a program to evaluate X-ray solution scattering of biological macromolecules from atomic coordinates. J. Appl. Cryst. 28, 768–773 (1995).

[b59] Bubb M. R. . Polylysine induces an antiparallel actin dimer that nucleates filament assembly: crystal structure at 3.5-Å resolution. J. Biol. Chem. 277, 20999–21006 (2002). 11932258 10.1074/jbc.M201371200

[b60] Rizvi S. A., Tereshko V., Kossiakoff A. A. & Kozmin S. A. Structure of bistramide alpha-actin complex at a 1.35 angstroms resolution. J. Am. Chem. Soc. 128, 3882–3883 (2006). 16551075 10.1021/ja058319c

