# Supplementary material for: Early nucleation events in the polymerization of actin, probed by time-resolved small-angle x-ray scattering
Source: Sci Rep. 2016 Oct 24;6:34539. doi: 10.1038/srep34539 (PMC5075782; doi:10.1038/srep34539)
Supplement: Supplementary Information [file srep34539-s1.pdf]

Supplementary information

Early nucleation events for polymerization of actin, probed by time-resolved small-angle x-ray scattering

Toshiro Oda[1], Tomoki Aihara[1] & Katsuzo Wakabayashi[2,1]

1) X-ray Structural Analysis Research Team, RIKEN SPring-8 Center, RIKEN Harima Institute, Kouto 1-1, Sayo, Hyogo 679-5148, Japan
2) Graduate School of Engineering Science, Osaka University, Toyonaka, Osaka 560-8531, Japan

Present Address
Toshiro Oda
Tokai Gakuin University, 5-68 Nakakirinocho, Kagamigahara, Gifu 504-8511, Japan

Tomoki Aihara
GE Healthcare Japan Corporation, Sanken Bidg., 3-25-1 Hyakunincho, Shinjuku, Tokyo 169-0073, Japan

Supplementary note

1. SAXS intensity profiles with liquid-like interference between particles.

In the formulation by Zernike and Prins [1,2], the SAXS intensity scattered with liquid-like interference between particles is written as,

$$I(q) = I_e(q)\overline{N} f^2(q)\left\{1 - \frac{1}{v_1}\int_0^\infty [1 - P(r)]\frac{\sin(qr)}{qr}4\pi r^2 dr\right\} \quad (1)$$

where q is the scattering vector length, $I_e(q)$ is the intensity scattered by one electron, $\overline{N}$, the average number of particles in the irradiated volume, v_1, the average volume per particle in the irradiated volume, $f(q)$, the spherically averaged scattering amplitude of a particle, and $P(r)$ is the probability distribution function of particles with an inter-particle separation, r. In the formulation, a square of the ensemble-average for the scattering amplitude of a particle, $\langle f(q)\rangle^2$ is put equal to an ensemble-average for a square of the scattering amplitudes, $\langle f^2(q)\rangle$ and is written by $f^2(q)$. We used the experimentally-obtained $I(q)/c\,|_{c=0}$ in place of $f^2(q)$.

$P(r)$ for a system of hard spherical particles is employed, where $P(r) = 0$ for $r < 2R$ (= a diameter) and $P(r) = 1$ for $r \geq 2R$. An interference peak calculated using this model is too broad to fit the experimental profiles. This is because the model has a sharp transition of $P(r)$ from zero to one in real space (see Figure 1). To fit the broad interference peak, we introduce a new parameter allowing for a gentle transition of $P(r)$. Here, the following $P(r)$ for the system of soft spherical particles is used,

$$P(r) = 1 - [1 + \exp\beta(r - r_0)]^{-1} \quad (2)$$

where r_0 is the closest approach distance between particles in the medium, and β is an adjustable parameter (see Figure 1). Using the Sommerfeld expansion for the integration, equation (1) becomes

$$\begin{aligned}
\frac{I(q)}{N^{bar} I_e(q)} &= \frac{I(q)}{c}\Big|_{c=0}[1 - f_1 - f_2] \\
f_1 &= \frac{4\pi}{q^3 v_1}[\sin(qr_0) - qr_0\cos(qr_0)] \\
f_2 &= \frac{2\pi^3}{3\beta^2 q v_1}[\sin(qr_0) + qr_0\cos(qr_0)]
\end{aligned} \quad (3)$$

where the term $I(q)/c|_{c=0}[1 - f_1]$ corresponds to an interference function for the system of hard spheres, which was derived by Debye [3]. The term $I(q)/c|_{c=0}[- f_2]$ is a correction factor for the contact between soft particles. Due to this term, the interference peak becomes sharper.

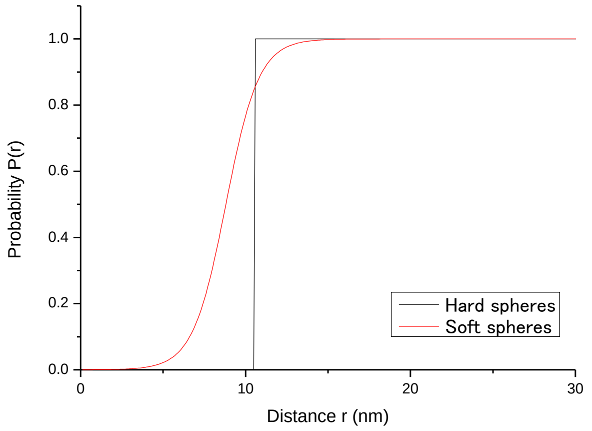

Figure 1 Probability distribution function *P*(*r*) of particles with an inter-particle separation, *r*.
$2R$ = 10.6 nm in the hard contact model. r_0 = 10.6 nm and β = 6 nm^{-1} in the soft contact model.

2. Description of time dependent changes of the average molecular weight in a nucleation-controlled polymerization

We employ the kinetic model of polymerization developed by Ferrone [4]. The principal assumption of this model is that all polymerization processes are sufficiently slow to allow equilibrium between nuclei and monomers. A typical diagram of free energy change (ΔG) of formation of various oligomers relative to the monomer state is as shown in Figure 2.

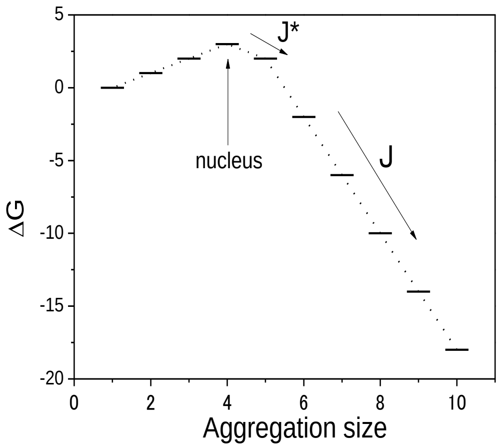

Figure 2 A schematic representation of the free energy change, ΔG, of oiligomer formation relative to the monomer. The abcissa represents an aggregation size (oligomer size).

When the concentration of nuclei is effectively low, they form a barrier to further growth. The rate of the formation of polymers is determined by the population of nuclei and the rate of elongation of the nuclei (the rate on crossing this barrier) J^* (= $k_+^* c^* - k_-^*$; k_+^* and k_-^* are the rate constants of elongation and dissociation of the nucleus, respectively). If c^* is the concentration of nuclei, polymers (the concentration, c_p) are then formed at the rate

$$\frac{dc_p}{dt} = J^* c^* \tag{4}$$

where $J*$ is the rate of elongation of the nucleus. Once polymers are formed, they add mass by accretion to their ends. If the monomer addition is done with the same rate J (= $k_+ c - k_-$: c is the concentration of monomers), independently of the polymer size, n, then the rate of the total concentration, Δ, of monomers that are incorporated into the polymers is written as

$$\frac{d\Delta}{dt} = Jc_p . \tag{5}$$

If all molecules are classed as either polymers or monomers, then putting the original concentration as c_0, we can write Δ at t,

$$\Delta(t) = c_0 - c(t). \tag{6}$$

By the use of a perturbation approach, the first-order solution to the set of equations has the form

$$\begin{aligned} \Delta(t) &= A[1-\cos(Bt)] \\ c_p &= (BA/J_0)\sin(Bt) \end{aligned} \tag{7}$$

where

$$A = J_0^* c_0^* \Big/ \frac{d(J^* c^*)}{dc}, \quad B^2 = J_0 \frac{d(J^* c^*)}{dc} \tag{8}$$

J_0 is a J at the concentration, c_0. A and B are parameters (see the paper of Ferrone [4]).

The weight-average molecular weights, M_{ave}, which are obtained by SAXS is written as,

$$M_{ave} = [\sum c_i M_i + (c_0 - \Delta)M] \Big/ c_0 = M\left(1\Big/ n_{tot} \sum_{i>n_c} i^2 n_i - 1\Big/ n_{tot} \sum_{i>n_c} i n_i + 1\right) \tag{9}$$

where M is the molecular weight of a monomer, and M_i, c_i and n_i are the molecular weight, the concentration and the number of the oligomer i, respectively, and n_c and n_{tot} are a size of the nucleus and the total number of molecules, respectively. The sums of in_i and i^2n_i in equation (9) at t are written approximately as

$$\sum_{i>n_c} in_i(t) \sim (B^2A/J_0)\int_0^t \cos(Bs)[(t-s)J_0+n_c+1]ds$$
$$\sum_{i>n_c} i^2n_i(t) \sim (B^2A/J_0)\int_0^t \cos(Bs)[(t-s)J_0+n_c+1]^2 ds \quad (10)$$

Using these equations, the time-dependence of the weight-average molecular weight is finally,

$$M_{ave}(t) = M\left(\frac{2AJ_0}{Bn_{tot}}\left[Bt-\sin(Bt)\right]+\frac{A(2n_c+1)}{n_{tot}}\left[1-\cos(Bt)\right]+\frac{BAn_c(n_c-1)}{J_0n_{tot}}\left[\sin(Bt)\right]+1\right)$$
$$\sim M\left(\frac{2AJ_0}{Bn_{tot}}\left[1/3!Bt^3\right]+\frac{A(2n_c+1)}{n_{tot}}\left[1/2!Bt^2\right]+\frac{BAn_c(n_c+1)}{J_0n_{tot}}\left[Bt\right]+1\right)$$
$$\sim M\left(\frac{B^2A}{n_{tot}}\frac{J_0}{3}t^3+\frac{B^2A}{n_{tot}}(2n_c+1)t^2+\frac{B^2A}{n_{tot}}\frac{n_c(n_c+1)}{J_0}t+1\right) \quad (11)$$
$$\sim M\left(\frac{\alpha\beta}{3}t^3+\alpha(2n_c+1)t^2+\frac{\alpha}{\beta}n_c(n_c+1)t+1\right)$$

in which we put $\alpha = B^2A/n_{tot}$ and $\beta = J_0$.

When n_c is equal to 4, equation (11) is written down by

$$M_{ave}(t) \sim M\left(\frac{\alpha\beta}{3}t^3+9\alpha t^2+20\frac{\alpha}{\beta}t+1\right). \quad (12)$$

3. Singular value decomposition (SVD) analysis

SAXS intensity data are collected row-wise in the matrix ***D***. The size of ***D*** is $r \times c$ (the number of rows times the number of columns) where r is the number of profiles and c is the number of representative points of the scattering vector length. To decide the number of the kind of complexes involved in the polymerization process, we calculate R = ***Rank*** (***D***). The rank of ***D*** is identical to the number of non-zero singular values obtained by SVD analysis. From the linear algebraic theorem, ***D*** is expressed by

$$\boldsymbol{D} = \boldsymbol{U}\boldsymbol{S}\,\boldsymbol{V}^{T} \tag{13}$$

where ***U*** and ***V*** are the orthonormal matrices: ***U*** consists of the eigenvectors of $\boldsymbol{D}\boldsymbol{D}^{\mathbf{T}}$, and ***V*** consists of the eigenvectors of $\boldsymbol{D}^{\mathbf{T}}\boldsymbol{D}$. ***S*** is a diagonal matrix of the singular values which are the positive square roots of the eigenvalues of $\boldsymbol{D}\boldsymbol{D}^{\mathbf{T}}$ and $\boldsymbol{D}^{\mathbf{T}}\boldsymbol{D}$. $\boldsymbol{V}^{\mathbf{T}}$and $\boldsymbol{D}^{\mathbf{T}}$ are the transpose matrices of ***V*** and ***D***, respectively.

Since the experimental data have some errors, we cannot decide whether the singular value, κ_j^2, is real zero or near zero. Thus, we cannot deduce explicitly the number of the kinds of complexes, n, during polymerization. To estimate n, we employed the following indicator (IND) function which has been proposed by Mallnowski [5,6].

$$IND = \frac{1}{(c-n)^2}\left[\frac{\sum_{j=n+1}^{j=c}\kappa_j^{\,2}}{r(c-n)}\right]^{1/2} . \tag{14}$$

The number n giving the minimum value of the IND function corresponds to the number of the kind of complexes in the polymerization process. The calculation was performed by the *svd* routine in the Scilab Package [7].

4. Isoscattering points

Assuming that all oligomer species scatter independently, the SAXS intensity profiles for a mixture of m different actin oligomers including monomers are described by [8],

$$I(q) = \sum_{l}^{m} c_l I_l(q) \tag{15}$$

where c_l is a weight concentration of the oligomer l, and $I_l(q)$ is a scattering intensity of the oligomer l per weight. $I_l(q)$ is written by [8] ,

$$I_l(q) = \frac{1}{l}\sum_{j}^{l} I_j(q) + \frac{2}{l}\left\langle \sum_{j}^{l-1}\sum_{k=j+1}^{l} \left|F_j(q)\right|\left|F_k(q)\right| \cos(qR_{jk} + \varphi_k - \varphi_j) \right\rangle \tag{16}$$

where R_{jk} is a distance between the subunit j with a form factor of $F_j(q)exp(i\varphi_j)$ and the subunit k with $F_k(q)$ $exp(i\varphi_k)$ which are included in the same oligomer l. < > denotes an orientational average. The first term corresponds to the scattering from individual actin subunits in each oligomer, and hence it has the same intensity profiles for all species. The second term accounts for interference between actin subunits in each oligomer. When the second terms for all species appearing during polymerization are zero at a fixed q, the profiles have an isoscattering point. In other words, all species must share a common periodicity in order to have an isoscattering point. When an actin subunit is approximated as a spherical molecule with a radius of R, the above equation [8] is,

$$I_l(q) = \frac{1}{l}\sum_{j}^{l} I_j(q) + \frac{2}{l}\sum_{j}^{l-1}\sum_{k=j+1}^{l} F_j(q) F_k(q) \frac{\sin(qR_{jk})}{qR_{jk}} \tag{17}$$

where $F(q)$ is the form factor of a sphere with a radius of R. To confirm the above consideration, we calculated the second term of equation (17) for a series of linear oligomers with the axial displacement of 5.52 nm and a series of helical oligomers having an F-actin type feature with the diameter of 16 nm, a 13 subunits/7 turns helical symmetry and an axial shift of 2.76 nm (see Figure 3). In the case of helical oligomers, the scattering profiles of the oligomers larger than the trimer give rise to a distinct isoscattering point. From the position of isoscattering point, the average nearest-neighbor distance of the subunits in the oligomers can be estimated. In the mixture of oligomers including monomers, dimers and trimers, the crossing point becomes blurred.

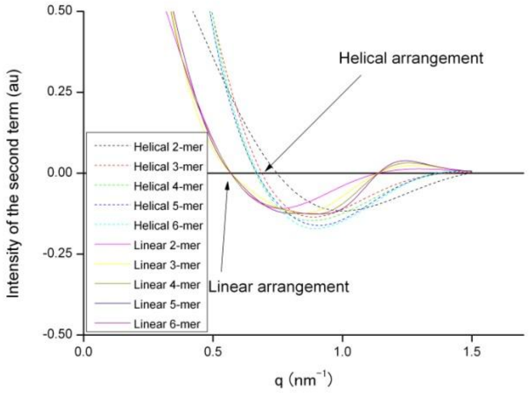

Figure 3 Intensity profiles of the interference term between the subunits in oligomers. Each arrow indicates the zero point of the interference term.

5. A kinetic model for actin polymerization

Our kinetic model for actin polymerization including the formation of nonpolymerizable dimer is formulated as follows.

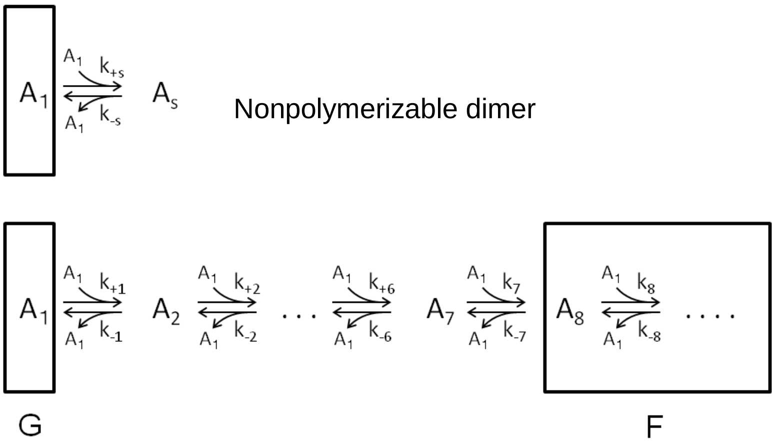

Figure 4 A sequential reaction scheme for actin polymerization including a nonpolymerizable dimer. A_1, A_s, A_i and F denote G-actin, nonpolymerizable dimer, intermediates comprising *i* actin molecules (*i*-mer) and F-actin, respectively. k_{s+} and k_{s-} are the rate constants of nonpolymerizable dimer formation and deformation, respectively, and k_{+i} and k_{-i} are the rate constants of the association and dissociation of a monomer for *i*-mer, respectively.

In the model, F-actin is defined as the complexes larger than an 8-mer (see Supplementary Figure 3), and the time-dependent SAXS intensities were calculated using the fractions of species present at a given time. In the growth phase of F-actin, k_{+i} = 5.5 μM $^{-1}$s^{-1} and k_{-i} = 1.8 s^{-1} were assumed. A_1, A_s and A_i are the number concentration of G-actin, nonpolymerizable dimer and *i*-mer, respectively, and w_i is the weight concentration of *i*-mer. Then the changes of A_s, A_2 and A_i with time and w_i are expressed as follows,

$$\frac{dA_s}{dt} = k_{+s}A_1^2 - k_{-s}A_s$$
$$\frac{dA_2}{dt} = k_{+1}A_1^2 - k_{-1}A_2 - k_{+2}A_2A_1 + k_{-2}A_3 \qquad (18)$$
$$\frac{dA_i}{dt} = k_{+(i-1)}A_{i-1}A_1 - k_{-(i-1)}A_i - k_{+i}A_iA_1 + k_{-i}A_{i+1}$$
$$w_i = iMA_i.$$

where *M* is the molecular mass of a monomeric actin.

We calculated the number concentration of *i*-mer up to 100-mer. Even if it is calculated up to 1000-mer, the result was the same. Using A_7, A_8 and A_9 thus obtained, the number concentration of F-actin (*N*) and the weight concentration of F-actin (*F*) together with that of monomers (A_1) were calculated as follows.

$$\frac{dN}{dt} = k_{+7}A_7A_1 - k_{-7}A_8$$
$$\frac{dF}{dt} = 8k_{+7}A_7A_1 - 8k_{-7}A_8 + k_{-8}A_9 + k_{+8}NA_1 - k_{-8}N \qquad (19)$$
$$\left(N = \sum_{i=8}^{\infty} A_i\right)$$

The change of A_1 with time is

$$\frac{dA_1}{dt} = -2k_{s+}A_1^2 - 2k_{+1}A_1^2 - k_{+7}A_7A_1 - \sum_{i=2}^{6} k_{+i}A_iA_1 - k_{+8}NA_1 + 2k_{-s}A_s + 2k_{-1}A_2 + \sum_{i=2}^{6} k_{-i}A_{i+1} + k_{-8}N \qquad (20)$$

These differential equations were solved using the *ode* command in the Scilab Package [7].

6. Explanation of a nucleus size in actin polymerization by the simple geometry of F-actin.

F-actin is constituted by two kinds of contacts between actin subunits with a longitudinal configuration along each strand (longitudinal contact) and a diagonal one between the two strands of F-actin (diagonal contact) [9]. By either one of the two kinds, a longitudinal dimer or a diagonal dimer is assumed to be formed. Upon the formation of helical trimer containing one turn of the basic helix of F-actin, the association of a monomer to the diagonal dimer simultaneously generates both the longitudinal and diagonal contacts likewise in the case of F-actin. As shown in Figure 5a, the diagram of the free energy change ΔG^0 in the pathway *via* the diagonal dimer would alter from the small slope ($d\Delta G^0/di$) to the large one at the dimer, and thus the nucleus in the downhill diagram is taken as the dimer by definition [10]. In contrast, in the pathway *via* the longitudinal dimer, two diagonal contacts are simultaneously generated. The diagram of the free energy change ΔG^0 would alter as shown in Figure 5b, and the nucleus is a trimer. Collectively, the formation of dimer or trimer as a nucleus has been deduced from the structural geometry of F-actin dependently on the strength of the two contacts [11]. In the normal condition, the longitudinal contact in F-actin is stronger than the diagonal contact, and a trimer is expected to be a nucleus.

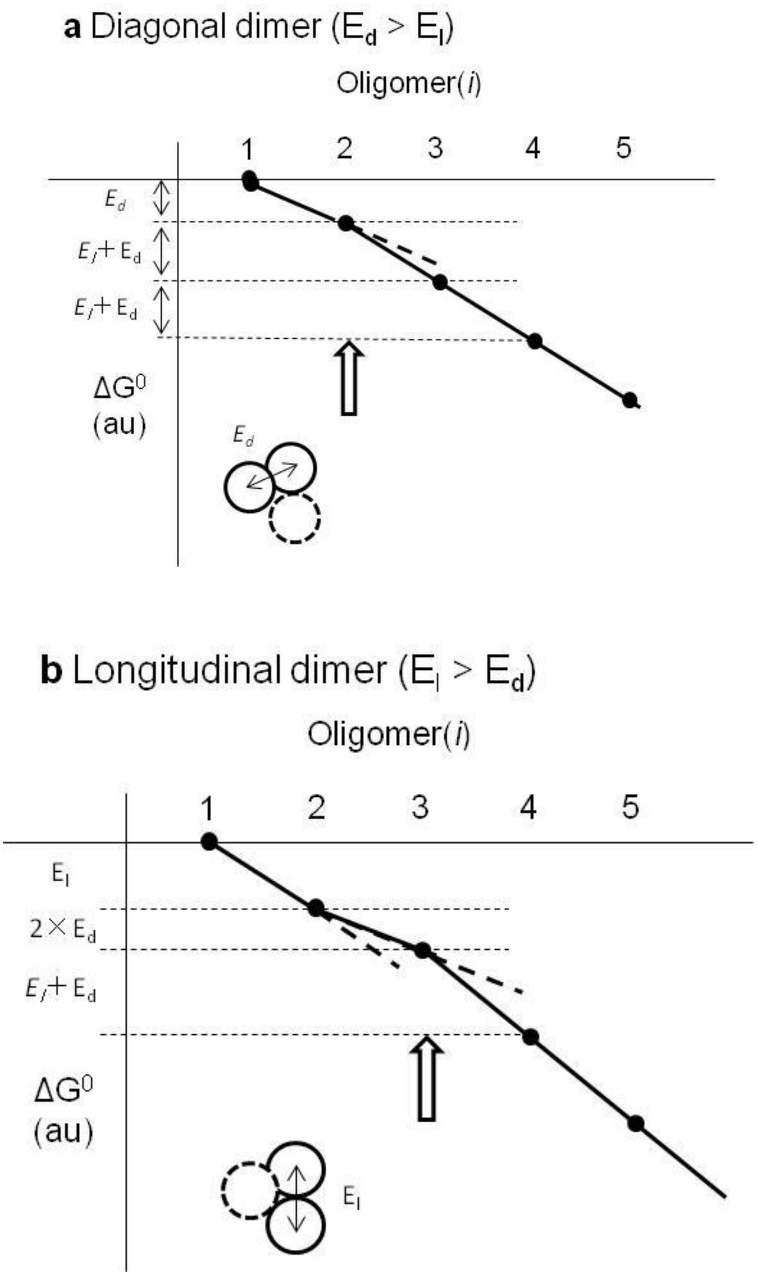

Figure 5 Schematic diagrams of free energy change versus oligomer size (*i*) *via* the diagonal and longitudinal dimers.

The free energy changes for the diagonal contact and the longitudinal contact are denoted by $-E_d$ and $-E_l$, respectively (E_d, E_l >0) . **a**, The path through the formation of diagonal dimer. The diagonal dimer is dominantly formed when the diagonal contact is stronger than the longitudinal one ($E_d > E_l$). In this diagram, the magnitude of free energy change for monomer addition upon the formation of each species is expressed: the diagonal dimer = $- E_d$, trimer = $- (E_l + E_d)$, tetramer = $- (E_l + E_d)$, pentamer = $- (E_l + E_d)$. **b**, The path through the formation of longitudinal dimer. The longitudinal dimer is formed at the condition of $E_l > E_d$. The free energy change for monomer addition upon the formation of each species is expressed: the longitudinal dimer = $- E_l$, trimer = $-2 \times E_d$ ($>- (E_l + E_d)$), tetramer = $- (E_l + E_d)$, pentamer = $- (E_l + E_d)$. Dashed lines in both graphs are the lines extrapolated from the line at the prior step.

Supplementary Figures

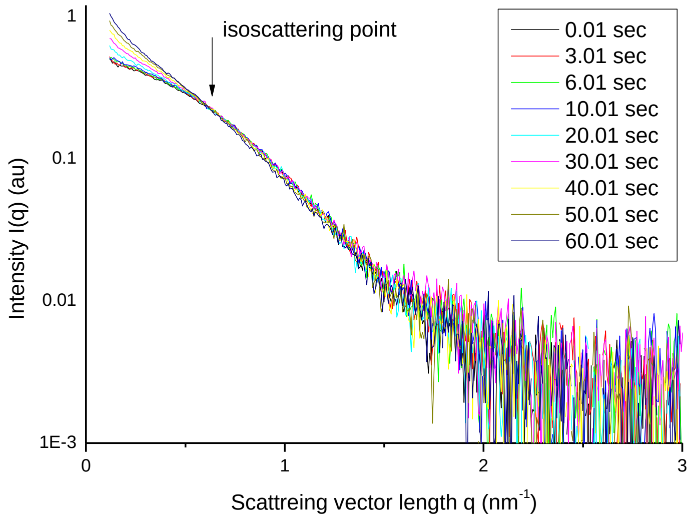

Supplementary Figure 1 A typical time series of SAXS intensity profiles during polymerization of actin. The numbers in inset box are time-elapse after mixing equal volumes of Mg-G-actin solution and salt solution at 10 ^{o}C and pH 8.0. Final concentrations after mixing were 31 μM for protein and 100 mM KCl and 1 mM $MgCl_2$ for salts.

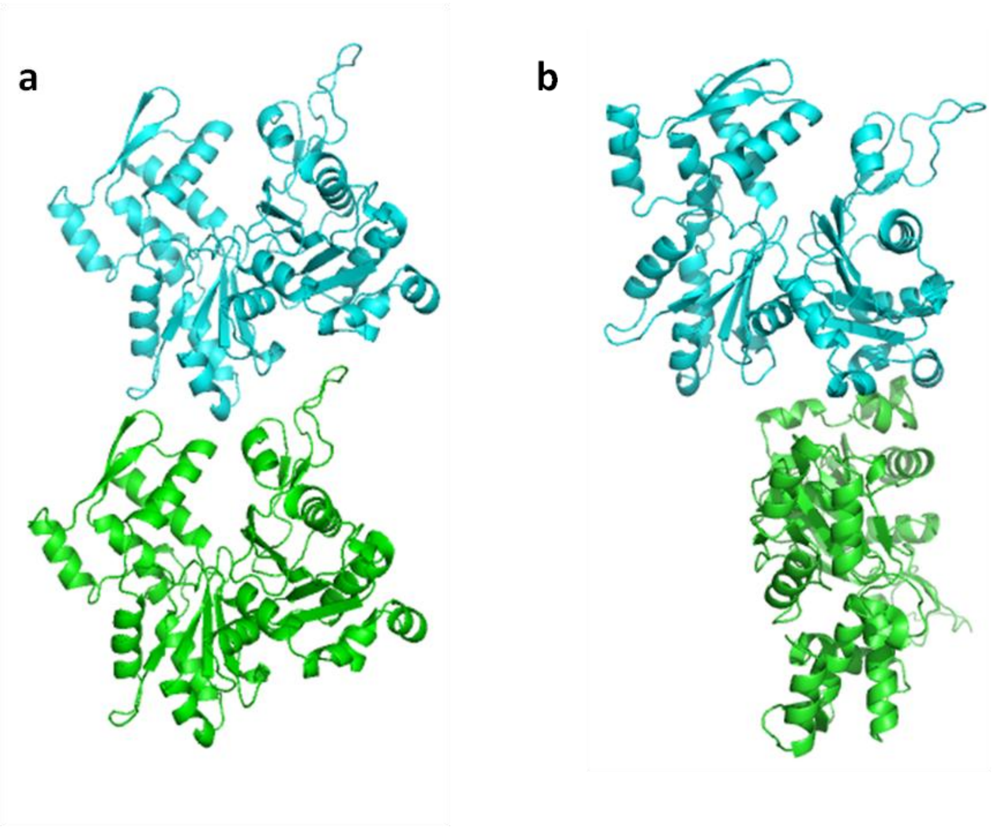

Supplementary Figure 2 Two kinds of actin dimer.

a, A parallel dimer. The dimer was made from the subunit arrangement along the crystal contacts of PDB code: 2FXU[12]. Theoretical SAXS intensity profile is indistinguishable from that of the dimer along the long-pitched helical strand of F-actin in the measured *q* range **b**, An anti-parallel dimer. The dimer was made from the arrangement of the anti-parallel dimer in the actin crystal of PDB code: 1LCU[13]. A similar dimer can be made from contacts in the actin crystal of PDB code: 2OAN[14].

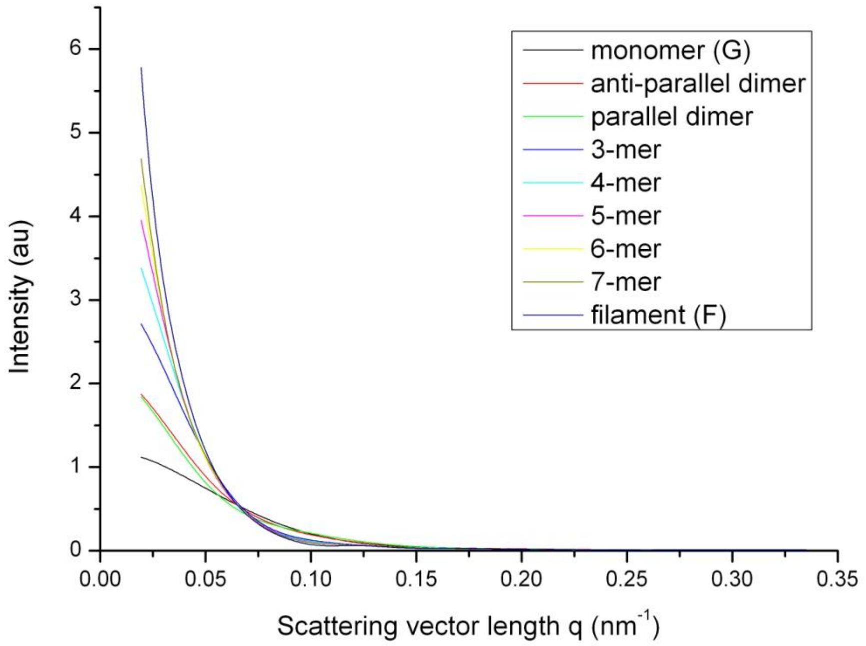

Supplementary Figure 3 SAXS intensity profiles calculated from G-actin, dimers, various intermediates and F-actin models.

The SAXS curves of oligomers larger than heptamer (7-mer) are indistinguishable from that of F-actin in the measured *q* range. The scattering profiles from oligomers larger than the trimer exhibit a distinct crossing point at q = 0.65 nm^{-1}.

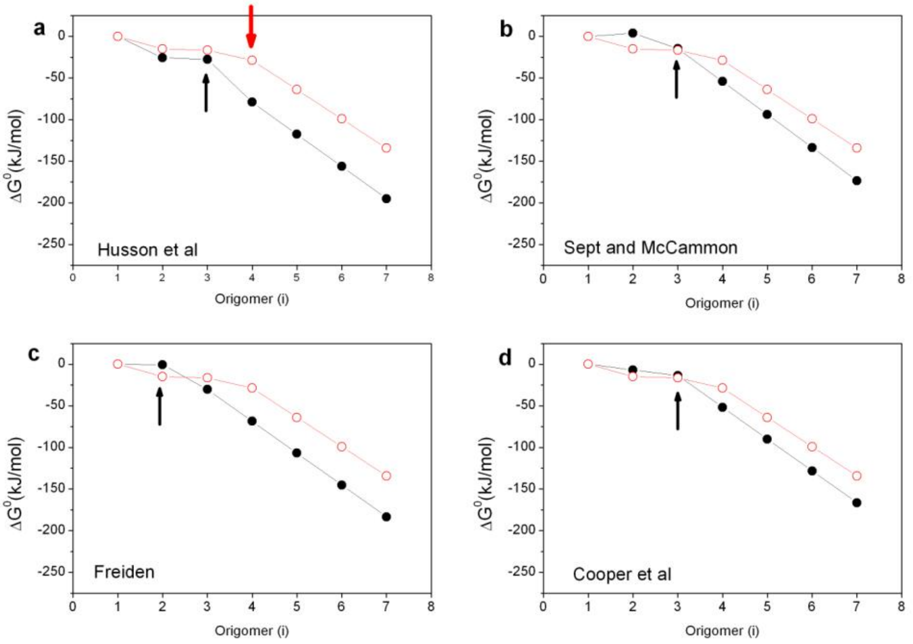

Supplementary Figure 4 Comparison of the profiles for standard free energy changes (ΔG^0) of *i* mer formation in the present work with those previously published.

Arrow in **a - d** shows oligomers of nucleus. **a**, The free energy change diagram by Husson *et al.* [15] (solid circles) is similar to that in the present work (open circles), but differs in the decrement upon the formation of tetramer (i = 4). The polymerization was done in the presence of Cordon-Bleu at room temperature. **b**, The free energy change diagram by Sept and McCammon [11] (solid circles). It was obtained from the kinetic and thermodynamic simulations of actin assembly without a conformational change of the subunit. The level of dimer is positive since in their simulation they did not include a decrease upon the monomer activation due to the addition of Mg-ions. Although the nucleus is assumed to be a dimer according to the definition of Ferrone [4,10] , by referring the effect of the monomer activation we mark the trimer at the flexing point of the slope as a nucleus. **c**, The free energy change diagram by Freiden [16] for Mg^{+}-induced polymerization at 20 ^{o}C and pH 8 (solid circles). The diagram is different from the present work, possibly due to the difference of salts used. **d**, The free energy change diagram by Cooper *et al.* [17] (solid circles). The free energy change upon monomer addition to dimer is assumed to be the same as upon monomer addition to trimer. The zero level in the diagrams of **c** and **d** was defined for activated monomers.

Polymerization of actin is divided into a nucleation phase and an elongation phase. The change from the nucleation to the elongation occurs at a flexing point in the free energy change diagram. We used the slow polymerization condition of the solution pH 8 and the temperature 10 ^{o}C. The two processes are strongly dependent on temperature and salt concentration. The balance between the two processes could affect the determination of nucleus size.

Alternative polymerization model: It is worth briefly mentioning that a bypass model in which a nucleus consisting of a tetramer is formed from two dimers can also account for the time-resolved SAXS data similarly to the sequential model we used in this study. It should be noted that the free energy levels of the dimer and the tetramer in the bypass model were identical to those in the sequential model with respect to monomer addition, and that the free energy diagram was also similar, and that the nucleus sizes are identical. The bypass model is impressive when the dimer can be assumed to be a longitudinal complex constituting of the flat conformation actin and the normal G-actin. A detailed discussion concerning the process from dimer to tetramer will be presented elsewhere.

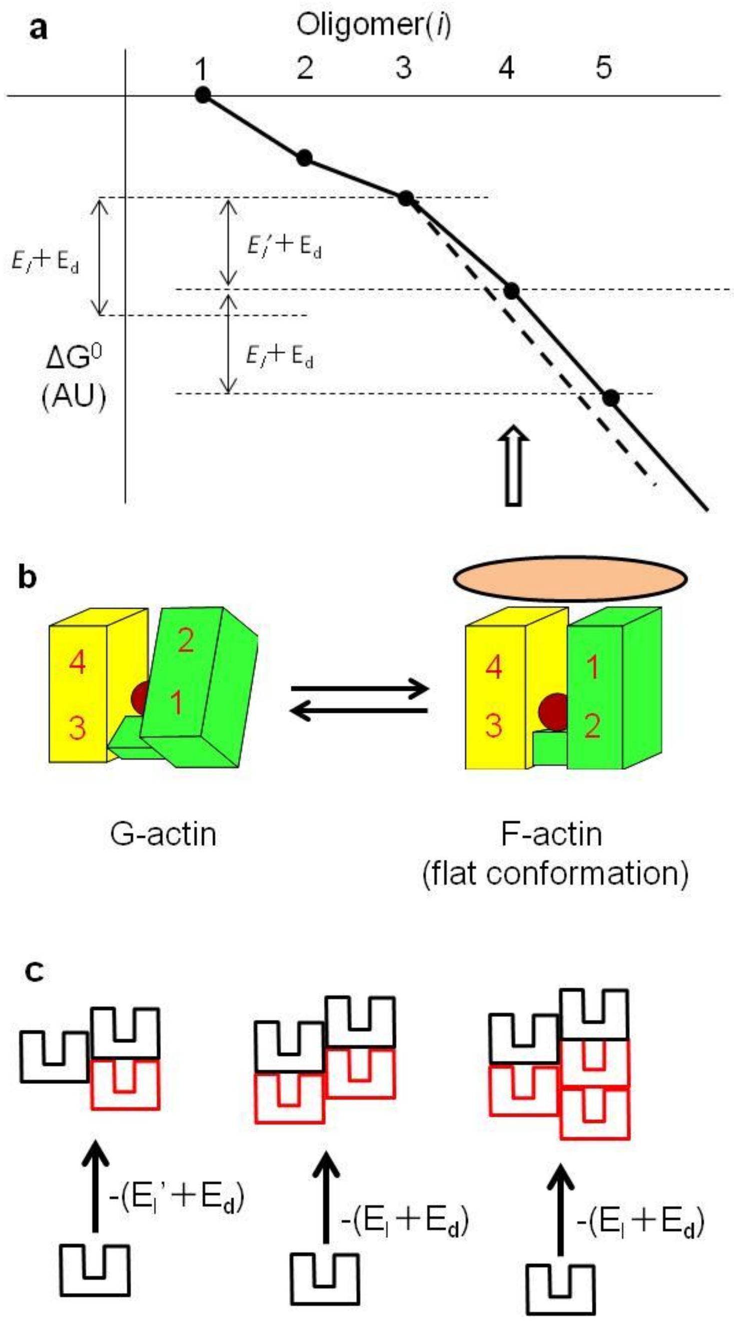

Supplementary Figure 5 Explanation of the diagram of the free energy change in the present work.
a, A schematic diagram of the standard free energy change versus oligomer size (*i*). **b** and **c**, the explanation of **a** by the simplified actin models [18,19]. In **c,** the red subunit is an actin subunit with a flat conformation and the black one is an actin subunit with the other conformation. In **a**, the magnitude of free energy change is defined as $-E_l$ for the longitudinal contact between actin subunits with a flat conformation (red in **c**), $-E_d$ for the diagonal contact between actin subunits with a flat conformation, $-E_l$' for the longitudinal contact between actin subunits with other conformation ($E_l' < E_l$), and $-E_d$' for the diagonal contact between actin subunits with other conformation ($E_d' < E_d$). Upon association of actin to the end of oligomer, the molecule is assumed to become a flat conformation. The solid line in **a** expresses the free energy changes with a distinction of the conformational difference of subunits; - ($E_l' + E_d$) upon monomer addition to helical trimer, - ($E_l + E_d$) upon monomer addition to tetramer and pentamer. The pentamer would a more stable, transient complex since the pentamer has one turn of the basic helix including three flat conformation subunits. The dashed line in **a** expresses the changes in the free energy without a distinction of the conformational difference of subunits (E_l' = E_l and E_d'= E_d).

Supplementary Table 1 Comparison of the rate constants (k^+ and k^-) and the equilibrium constants (K). The values are the averages for 9 sets giving the lowest χ^2 -values, which were obtained from the different initial sets of parameters (see text).

	In this study			
	k+ ($\mu M^{-1}s^{-1}$)	k- (s^{-1})	K^{-1} (μM)	ΔG(kJ/mol)
monomer -> anti*-dimer (K_1)	1.5×10^{-4}	2.4×10^{-2}	1.6×10^{2}	-21
monomer -> p**-dimer (K_s)			1.7×10^{3}	-15
dimer -> trimer (K_2)			5.5×10^{5}	-1.4
trimer -> tetramer (K_3)	1.7×10^{-1}	8.8×10^{2}	5.2×10^{3}	-12
tetramer -> pentamer (K_4)	5.5	1.8	0.31	-35
canonical growth (Cc)	5.5	1.8	0.31	-35

*anti-parallel, **parallel

The individual rate constants for the formation of dimer and trimer were unable to be uniquely decided probably because the steps were in steady state equilibrium [20] and only the equilibrium constants ($K_i = k_{+i}/k_{-i}$) could be determined; the values of K_1 and $K_1K_2K_3$ were unique. However, K_2 giving low χ^2-values distributes around two values dependently on the fraction of trimer, and the parameters of model exhibiting no visible amount of trimer as suggested by the AIC analysis were finally decided.

The value of (elongation rate constant) × (nucleation rate constant), which has frequently been used as a fitting parameter in analysis of polymerization [20,21] was calculated to be 1.0×10^{15} $M^{-4}s^{-2}$ (= $k_{+4}K_1K_2k_{+3}$) from these rate constants. This was comparable to 3.96×10^{15} $M^{-4}s^{-2}$ in Tobacman and Korn [21], and (0.984 - 2.250) $\times 10^{15}$ $M^{-4}s^{-2}$ in Fesce *et al* [22].

References

1 Zernike, F. & Prins, J. A. Die Beugung von Röntgenstrahlen in Flüssigkeiten als Effekt der Molekülanordnung *Z. Phys.* **41**, 184-194 (1927).

2 Guinier, A. *X-ray Diffraction in Crystals, Imperfect Crystal, and Amorphous Bodies.* Ch. 3, 63-72(W. H. Freeman and Company, 1963).

3 Debye, P. Uber die Zerstreuung von Rontgenstrahlen an amorphen Korpern. *Phys. Z.* **28**, 135 -141 (1927).

4 Ferrone, F. Analysis of protein aggregation kinetics. *Methods Enzymol.* **309**, 256-274 (1999).

5 Mallnowski, E. R. Determination of the number of factors and the experimental error in a data matrix. *Anal. Chem.* **49**, 612-617 (1977).

6 Mallnowski, E. R. Theory of error in factor analysis. *Anal. Chem.* **49**, 606-612 (1977).

7 Scilab: Free and open source software for numerical computation (OS, Version 5.22) [Software]. Available from: http://www.scilab.org (2012).

8 Porod, G. *General Theory* in *Small Angle X-ray Scattering* (eds O. Glatter & O. Kratky) Ch. 2, 17-51 (Academic Press, 1982).

9 Holmes, K. C., Popp, D., Gebhard, W. & Kabsch, W. Atomic model of the actin filament. *Nature* **347**, 44-49 (1990).

10 De Greef, T. F. A. *et al.* Supramolecular polymerization. *Chem. Rev.* **109**, 5687-5754 (2009).

11 Sept, D. & McCammon, J. A. Thermodynamics and kinetics of actin filament nucleation. *Biophys. J.* **81**, 667-674 (2001).

12 Rizvi, S. A., Tereshko, V., Kossiakoff, A. A. & Kozmin, S. A. Structure of bistramide α-actin complex at a 1.35 angstroms resolution. *J. Am. Chem. Soc.* **128**, 3882-3883 (2006).

13 Bubb, M. R. *et al.* Polylysine induces an antiparallel actin dimer that nucleates filament assembly: crystal structure at 3.5-Å resolution. *J. Biol. Chem.* **277**, 20999-21006 (2002).

14 Lassing, I. *et al.* Molecular and structural basis for redox regulation of β-actin. *J. Mol. Biol.* **370**, 331-348 (2007).

15 Husson, C., Renault, L., Didry, D., Pantaloni, D. & Carlier, M. F. Cordon-bleu uses WH2 domains as multifunctional dynamizers of actin filament assembly. *Mol. Cell* **43**, 464-477 (2011).

16 Frieden, C. Polymerization of actin: mechanism of the Mg^{2+}-induced process at pH 8 and 20 °C. *Proc. Natl. Acad. Sci. USA* **80**, 6513-6517 (1983).

17 Cooper, J. A., Buhle, E. L., Jr., Walker, S. B., Tsong, T. Y. & Pollard, T. D. Kinetic evidence for a monomer activation step in actin polymerization. *Biochemistry* **22**, 2193-2202 (1983).

18 Oda, T., Iwasa, M., Aihara, T., Maeda, Y. & Narita, A. The nature of the globular- to fibrous-actin transition. *Nature* **457**, 441-445 (2009).

19 Oda, T. & Maeda, Y. Multiple Conformations of F-actin. *Structure* **18**, 761-767 (2010).

20 Wegner, A. & Engel, J. Kinetics of the cooperative association of actin to actin filaments. *Biophys. Chem.* **3**, 215-225 (1975).

21 Tobacman, L. S. & Korn, E. D. The kinetics of actin nucleation and polymerization. *J. Biol. Chem.* **258**, 3207-3214 (1983).

22 Fesce, R., Benfenati, F., Greengard, P. & Valtorta, F. Effects of the neuronal phosphoprotein synapsin I on actin polymerization. II. Analytical interpretation of kinetic curves. *J. Biol. Chem.* **267**, 11289-11299 (1992).
